# SPSB1‐mediated inhibition of TGF‐β receptor‐II impairs myogenesis in inflammation

**DOI:** 10.1002/jcsm.13252

**Published:** 2023-05-20

**Authors:** Yi Li, Niklas Dörmann, Björn Brinschwitz, Melanie Kny, Elisa Martin, Kirsten Bartels, Ning Li, Priyanka Voori Giri, Stefan Schwanz, Michael Boschmann, Susanne Hille, Britta Fielitz, Tobias Wollersheim, Julius Grunow, Stephan B. Felix, Steffen Weber‐Carstens, Friedrich C. Luft, Oliver J. Müller, Jens Fielitz

**Affiliations:** ^1^ Experimental and Clinical Research Center (ECRC), Charité‐Universitätsmedizin Berlin Max Delbrück Center (MDC) for Molecular Medicine in the Helmholtz Association Berlin Germany; ^2^ DZHK (German Center for Cardiovascular Research), partner site Greifswald Greifswald Germany; ^3^ Department of Internal Medicine B, Cardiology University Medicine Greifswald Greifswald Germany; ^4^ Department of Internal Medicine III University of Kiel Kiel Germany; ^5^ German Centre for Cardiovascular Research (DZHK), partner site Hamburg/Kiel/Lübeck Germany; ^6^ Department of Anaesthesiology and Operative Intensive Care Medicine (CCM, CVK), Charité ‐ Universitätsmedizin Berlin, corporate member of Freie Universität Berlin Humboldt‐Universität zu Berlin and Berlin Institute of Health Berlin Germany

**Keywords:** Critical illness myopathy, Sepsis, Inflammation‐induced muscle atrophy, SPSB1, TGFβ receptor II, Myogenic differentiation

## Abstract

**Background:**

Sepsis‐induced intensive care unit‐acquired weakness (ICUAW) features profound muscle atrophy and attenuated muscle regeneration related to malfunctioning satellite cells. Transforming growth factor beta (TGF‐β) is involved in both processes. We uncovered an increased expression of the TGF‐β receptor II (TβRII)‐inhibitor SPRY domain‐containing and SOCS‐box protein 1 (SPSB1) in skeletal muscle of septic mice. We hypothesized that SPSB1‐mediated inhibition of TβRII signalling impairs myogenic differentiation in response to inflammation.

**Methods:**

We performed gene expression analyses in skeletal muscle of cecal ligation and puncture‐ (CLP) and sham‐operated mice, as well as *vastus lateralis* of critically ill and control patients. Pro‐inflammatory cytokines and specific pathway inhibitors were used to quantitate *Spsb1* expression in myocytes. Retroviral expression plasmids were used to investigate the effects of SPSB1 on TGF‐β/TβRII signalling and myogenesis in primary and immortalized myoblasts and differentiated myotubes. For mechanistical analyses we used coimmunoprecipitation, ubiquitination, protein half‐life, and protein synthesis assays. Differentiation and fusion indices were determined by immunocytochemistry, and differentiation factors were quantified by qRT‐PCR and Western blot analyses.

**Results:**

*SPSB1* expression was increased in skeletal muscle of ICUAW patients and septic mice. Tumour necrosis factor (TNF), interleukin‐1β (IL‐1β), and IL‐6 increased the *Spsb1* expression in C2C12 myotubes. TNF‐ and IL‐1β‐induced *Spsb1* expression was mediated by NF‐κB, whereas IL‐6 increased the *Spsb1* expression via the glycoprotein 130/JAK2/STAT3 pathway. All cytokines reduced myogenic differentiation. SPSB1 avidly interacted with TβRII, resulting in TβRII ubiquitination and destabilization. SPSB1 impaired TβRII‐Akt‐Myogenin signalling and diminished protein synthesis in myocytes. Overexpression of SPSB1 decreased the expression of early (*Myog*, *Mymk*, *Mymx*) and late (*Myh1*, *3*, *7*) differentiation‐markers. As a result, myoblast fusion and myogenic differentiation were impaired. These effects were mediated by the SPRY‐ and SOCS‐box domains of SPSB1. Co‐expression of SPSB1 with Akt or Myogenin reversed the inhibitory effects of SPSB1 on protein synthesis and myogenic differentiation. Downregulation of *Spsb1* by AAV9‐mediated shRNA attenuated muscle weight loss and atrophy gene expression in skeletal muscle of septic mice.

**Conclusions:**

Inflammatory cytokines via their respective signalling pathways cause an increase in SPSB1 expression in myocytes and attenuate myogenic differentiation. SPSB1‐mediated inhibition of TβRII‐Akt‐Myogenin signalling and protein synthesis contributes to a disturbed myocyte homeostasis and myogenic differentiation that occurs during inflammation.

## Introduction

Septic patients often develop intensive care unit‐acquired weakness (ICUAW), which is accompanied by muscle‐mass loss, increased morbidity, and mortality.^1^, ^2^ ICUAW is defined as ‘clinically detected weakness in critically ill patients in whom there is no plausible aetiology other than critical illness’.^3^ Patients with ICUAW are classified into those with critical illness myopathy (CIM), critical illness polyneuropathy (CIP), or a combination of both. In sepsis, inflammatory cytokines, such as interleukin‐6 (IL‐6), IL‐1β, tumour necrosis factor (TNF), and the acute phase protein, serum amyloid A1 (SAA1), are increased in serum and skeletal muscle of patients and mice.^4^, ^5^ This causes an activation of protein degradation by the ubiquitin‐proteasome system (UPS) and an inhibition of protein synthesis in skeletal muscle of patients.^6^ We reported that by inhibiting cytokine signalling muscle atrophy in sepsis can be reduced, but not abrogated^4^, ^5^ and reasoned that additional pathways must be operative, because ICU survivors still have impaired muscle function even 5 years after their illness.^7^ This might be due to an impaired regenerative capacity of muscle in ICU patients^8^, ^9^ and septic mice^10^ even though a regenerative capacity via muscle residing stem cells, known as satellite cells, would be expected to be operative.^11^ Satellite cells are required for skeletal muscle growth and regeneration. Once activated, satellite cells undergo myogenic differentiation to form myofibers and proliferate to replenish the satellite cell pool in muscle. Orderly myogenesis requires the sequential expression of myogenic transcription factors, myoblast‐fusion proteins^12^, ^13^ and contractile elements.^14^ However, the importance of myogenesis in critical illness is not well understood. Recently, an activation of Transforming growth factor beta (TGF‐β) signalling that is involved in myogenesis and elevated TGF‐β receptor type II (TβRII) levels were reported in muscle of critically ill patients,^15^ suggesting their involvement in ICUAW. TGF‐β binds to the TβRI and TβRII complex to activate both, canonical Smad‐dependent, and non‐canonical signalling pathways, such as Akt that activates protein synthesis and muscle growth.^16^ Importantly, TGF‐β signalling can be inhibited by the splA/ryanodine receptor (SPRY) domain and SOCS‐box domain 1 (SPSB1/*Spsb1*) protein that targets TβRII for UPS‐dependent degradation in neuroblastoma cells.^17^ Because we found SPSB1 to be strongly increased in muscle of ICUAW patients and septic mice we hypothesized that SPSB1‐mediated inhibition of TβRII‐signalling impairs protein homeostasis and subsequently myogenesis in response to inflammation, which may contribute to the impaired regenerative capacity of muscle in ICUAW patients.

## Methods

### Patient samples

The institutional review board of the Charité University Medicine Berlin, Germany, approved the study, and written informed consent was obtained from the patients or their legal proxy (Charité EA2/061/06; ISRCTN77569430). We analysed gene expression in *vastus lateralis* muscle biopsy specimens from patients prone to develop ICUAW; these patients (*n* = 7) were critically ill, mechanically ventilated with a SOFA‐score ≥8 on three consecutive days within the first 5 days after ICU admission. Muscle biopsy specimens from healthy patients (*n* = 12) were used as controls (NCT 01468220). For more details on clinical data, please refer to Wollersheim et al.^6^

### Animal models

All animal procedures were approved by the Landesamt für Gesundheit und Soziales (Berlin, Germany; #G207/13) or the Landesamt für Landwirtschaft, Lebensmittelsicherheit und Fischerei (Rostock, Germany, #7221.3‐1‐074/20). The investigation conforms to the *Guide for the Care and Use of Laboratory Animals* published by the US National Institutes of Health (Publication #85‐23, revised 1985), as well as the German Law on the Protection of Animals. Cecal ligation and puncture (CLP) surgery was performed in 8‐week‐old male B6(C)/Rj‐Tyr^c/c^ and 12‐ to 16‐week‐old male C57BL/6 J, *Nlrp3* knockout and *Nlrp3* wildtype mice, as indicated and recently described.^4^, ^5^, ^18^ Mice were sacrificed 24 and 96 h after surgery, and *tibialis anterior* (TA), *gastrocnemius/plantaris* (GP), *extensor digitorum longus* (EDL), and *soleus* (Soleus) muscles were harvested for analyses. For further details on experimental procedures, please refer to the supporting information.

### Statistics

All experiments were performed independently and at least three times using biological triplicates each. Statistical analysis was performed using GraphPad Prism7 (GraphPad Software, Inc., USA). Differences between two groups were evaluated with unpaired two‐tailed Student's *t*‐test. One‐way analysis of variance (ANOVA) followed by Tukey's post‐hoc test was used for comparison of more than two independent groups with only one factor. For two factors two‐way ANOVA followed by Tukey's post‐hoc test was used. Data are presented as mean ± standard deviation. Photoshop (Adobe, USA) and Illustrator (Adobe, USA) and FIJI/ImageJ software (Wayne Rasband, National Institutes of Health USA) were used for plots. *P* < 0.05 was considered statistically significant.

## Results

### SPSB1 is upregulated in inflammation‐induced skeletal muscle atrophy

**Figure 1:**
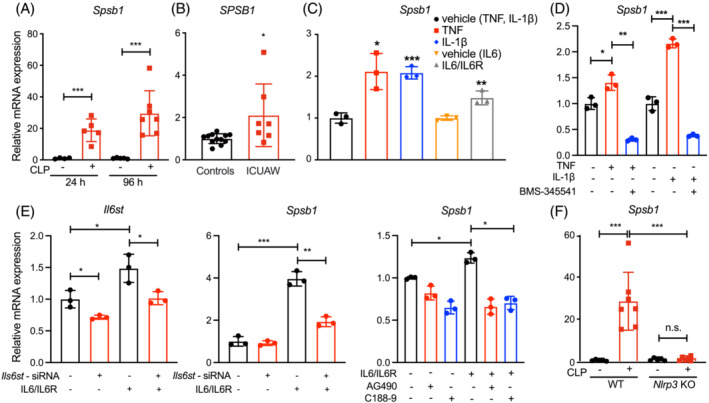
*Spsb1* is upregulated in inflammation‐induced skeletal muscle atrophy. (A) Quantitative RT‐PCR (qRT‐PCR) analysis of *Spsb1* from the tibialis anterior (TA) muscle in 12‐week‐old male C57BL/6J mice subjected to cecal ligation and puncture (CLP, *n* = 5, 24 h; *n* = 7, 96 h) or sham surgery (sham, *n* = 4, 24 h; *n* = 5, 96 h). (B) qRT‐PCR of *SPSB1* from the *vastus lateralis* muscle of patients with intensive care unit‐acquired weakness (ICUAW, *n* = 7) compared with healthy subjects (controls, *n* = 12). (C) qRT‐PCR of *Spsb1* from five‐day‐differentiated C2C12 myotubes (MT5) treated with TNF (10 ng/mL), IL‐1β (10 ng/mL) or IL6/IL6R (100 ng/mL) for 2 h. (D) qRT‐PCR of *Spsb1* from MT5 that were pretreated with the IKK1‐inhibitor BMS‐345541 (5 μM) 60 min before TNF or IL‐1β treatment. (E) qRT‐PCR of *Il1st* and *Spsb1* from MT5 after *Il6st*‐siRNA transfection and pretreatment with the JAK2 inhibitor AG490 or the STAT3 inhibitor C188–9 prior to IL‐6 treatment. (F) qRT‐PCR analysis of *Spsb1* from the TA muscle of *Nlrp3* knockout (KO) and *Nlrp3* wildtype (WT) littermate control mice subjected to CLP (*n* = 7) or sham (*n* = 5) surgery for 96 h, as indicated. mRNA expression was normalized to *Gapdh*. Data in panels (A) and (D–F) were analysed with two‐way ANOVA followed by Tukey's post‐hoc test; data in panel (B) were analysed with two‐tailed Student's *t*‐test; data in panel (C) were analysed with one‐way ANOVA followed by Tukey's post‐hoc test. *P < 0.05, **P < 0.01, ***P < 0.001. n.s. denotes not significant.

To identify genes involved in impaired regenerative capacity in muscle during sepsis, we performed next generation sequencing of RNAs (RNAseq) isolated from TA muscles of CLP and sham operated male wildtype (WT) mice (for details, please see Zanders et al.^18^). Besides the atrophy markers *Trim63*/MuRF1, *Fbxo32*/Atrogin‐1, and *Fbxo30*/MuSA1 (all induced >5‐fold by sepsis, *P* < 0.001) the expression of *Spsb1* was significantly increased after 24 and 96 h of sepsis (24 h: 20‐fold; 96 h: 34‐fold, *P* < 0.001). qRT‐PCR confirmed an increased *Spsb1* expression in TA of septic mice 24 and 96 h after surgery (Figure 1A). *Spsb1* was also increased in GP, Soleus and EDL 24 h, and in TA and GP but not Soleus or EDL 96 h after surgery (Figure S1). Because fast twitch/type‐II myofibers show the strongest atrophy response in sepsis,^6^ we analysed if these fibres contain higher amounts of SPSB1. Immunofluorescent staining of histological cross‐sections from TA revealed that SPSB1 was enriched in type‐IIa myofibers (Figure S2). Because the SPSB‐protein family is composed of four well‐conserved members (SPSB1, SPSB2, SPSB3, SPSB4),^19^ we investigated if their expression is also affected by sepsis. *Spsb2* was increased in Soleus and EDL 24 and 96 h after surgery, respectively, and *Spsb4* remained unchanged. Interestingly, *Spsb3* expression was increased in all muscles but only 96 h after CLP surgery (Figure S1). Our data show that *Spsb1* expression is increased in muscles of septic mice and shows a distinct expression pattern within the SPSB‐family. We next quantitated the *SPSB1* expression in muscle of ICUAW patients and found it to be increased when compared with healthy controls (Figure 1B). We also observed an increased *SPSB2* and *SPSB3*, but not *SPSB4* expression in muscle of ICUAW patients (Figure S3).

To investigate if *Spsb1* is expressed in myocytes and if inflammatory cytokines regulate its expression, we treated 5‐days‐differentiated myotubes (MT5) with TNF, IL‐1β, and IL‐6/IL‐6R for different time points from 1 to 72 h (Figure S4A), respectively, and measured *Spsb1* expression. We also treated MT5 with SAA1 and LPS for 72 h. All inflammatory cytokines, SAA1 and LPS increased *Spsb1* expression in MT5 (Figure S4A,B). The highest increase in *Spsb1* expression was observed after 2 h of cytokine treatment (Figure 1C), which was used for further analyses. We treated differentiating C2C12 myoblasts with TGF‐β and found it to also increase *Spsb1* expression after 24 and 72 h (Figure S4C). We next investigated the signalling pathways involved in cytokine‐induced *Spsb1* expression. TNF‐ and IL‐1β‐induced *Spsb1* expression was inhibited by the IκB kinase‐inhibitor BMS‐345541 (Figure 1D) indicating that this effect was mediated by NF‐κB. To investigate how IL‐6 increased *Spsb1* expression we reduced the IL‐6 receptor glycoprotein (gp) 130, encoded by *Il6st*, by siRNA prior to IL‐6 treatment in MT5, which attenuated IL‐6‐induced *Spsb1* expression (Figure 1E). The JAK2 inhibitor AG490 and the STAT3 inhibitor C188‐9 also attenuated IL‐6‐induced *Spsb1* expression (Figure 1E) indicating that the gp130/JAK2/STAT3 pathway mediated this effect. We previously showed that IL‐1β serum levels and muscular *Il6* expression were reduced in septic *Nlrp3* KO mice, and that these mice are protected from muscle atrophy in sepsis.^4^ In accordance with those findings, *Spsb1* expression increased in TA muscle of septic *Nlrp3* WT but not in *Nlrp3* KO mice (Figure 1F), further supporting that proinflammatory cytokines, especially IL‐1β, increase muscular *Spsb1* expression. In summary, our data show that inflammation *in vivo* and proinflammatory cytokines *in vitro* cause an increase in *Spsb1* expression in muscle and myocytes, respectively.

### SPSB1 interacts with and increases the turnover of TGF‐β receptor II

SPSB1 is a cullin‐5 E3 ubiquitin ligase adaptor^20^ and associates with and degrades the TβRII in non‐muscle cells.^17^ To analyse changes in TβRII in muscle during sepsis, we performed immunofluorescent staining of histological cross sections from TA of sham‐ and CLP‐operated mice 96 h after surgery. We found that TβRII was ubiquitously expressed in all myofibers and was localized to the sarcolemma in sham‐operated mice. In contrast, membranous TβRII‐staining was reduced in myofibers of CLP mice (Figure S5) suggesting an association between increased *Spsb1* expression and a decrease in its putative target TβRII in myofibers during sepsis. Consistently, our RNAseq data showed a significant downregulation of genes contained in the Gene Ontology (GO) term ‘cellular response to transforming growth factor beta stimulus’ (GO:0071560, *P* = 8.84*10^−5^, FDR 0.002; Figure S6) and the Kyoto Encyclopedia of Genes and Genomes (KEGG)‐pathway ‘TGF‐beta signaling pathway’ (mmu04350, *P* = 5.4*10^−4^, FDR 0.0038, Figure S7A,B).

**Figure 2:**
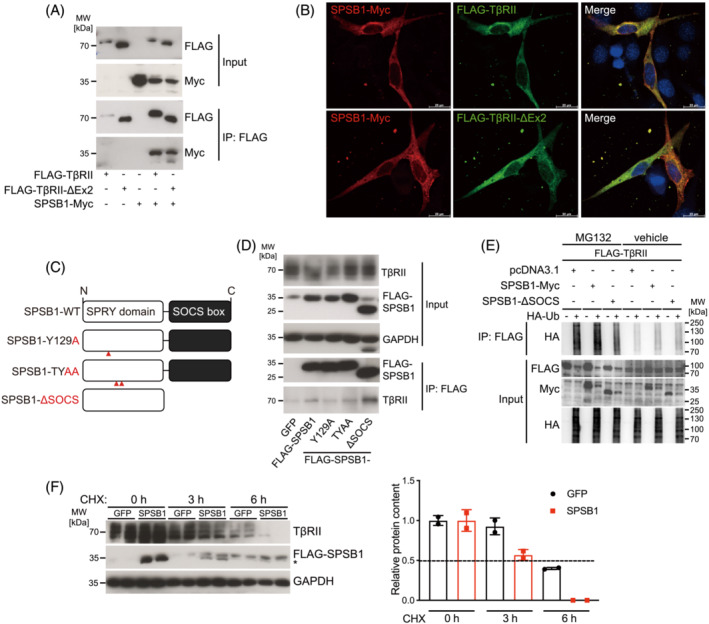
SPSB1 associates with and ubiquitinates TGF‐β receptor II and reduces its stability. (A) Co‐immunoprecipitation (Co‐IP) using lysates from C2C12 cells transfected with FLAG‐TβRII, FLAG‐TβRII‐ΔEx2 and SPSB1‐Myc. Extracts were immunoprecipitated (IP) with anti‐FLAG agarose and detected with antibodies against Myc and FLAG, as indicated. Input Western blot with anti‐Myc and anti‐FLAG antibody as indicated. (B) Immunofluorescence using anti‐FLAG antibody together with A488‐coupled secondary antibody (green) and anti‐Myc antibody together with A555‐coupled secondary antibody (red) to detect SPSB1‐Myc, FLAG‐TβRII and FLAG‐TβRII‐ΔEx2 in transfected C2C12 cells. Nuclei were stained with DAPI (blue). Scale bar, 20 μm. (C) Pictogram of functional domains and mutation sites of SPSB1 constructs. (D) Co‐IP of FLAG‐SPSB1 (WT) and mutants (SPSB1‐Y129A, ‐TYAA or ‐ΔSOCS) with endogenous TβRII from MT5 C2C12 cells overexpressing GFP, SPSB1 and SPSB1 mutants. Extracts were IP with anti‐FLAG agarose and detected with anti‐TβRII antibody. Input proteins were detected with anti‐FLAG, TβRII and GAPDH antibodies as indicated. (E) COS‐7 cells were transfected with FLAG‐TβRII, SPSB1‐Myc, SPSB1‐ΔSOCS and HA‐Ub, as indicated. Cells were treated with MG132 (25 μM) or vehicle (DMSO 0.25%) 42 h post‐transfection for a further 6 h. ll cells were lysed 48 h post‐transfection and lysates were immunoprecipitated (IP) with anti‐FLAG affinity gel. Immunoblotting (IB) with indicated antibodies. (F) Cells were infected with a retrovirus encoding GFP or SPSB1 for 48 h and then treated with cycloheximide (CHX, 50 μg/mL) for indicated timepoints. Anti‐FLAG antibody shows overexpressed FLAG‐SPSB1; specific band is indicated by asterisk. GAPDH was used as loading control. Densitometric analysis; dotted line indicates 50% abundance of TβRII. *N* = 3 biologically independent experiments; representative blots are shown. Data are presented as mean ± standard deviation.

We next investigated if SPSB1 regulates TβRII function in myocytes. We identified two transcript variants of TβRII, full‐length TβRII and TβRII devoid of exon2 (TβRII‐ΔEx2) in myocytes. Using co‐immunoprecipitation analyses we found that SPSB1 physically interacted with both TβRII and TβRII‐ΔEx2 to a similar degree (Figure 2A). SPSB1 and both TβRII variants were localized to the cytoplasm and the membrane of C2C12 cells (Figure S8A), where they also co‐localized (Figure 2B). Endogenous TβRII and overexpressed SPSB1 colocalized in C2C12 myoblasts (Figure S8B). To investigate if the splA/ryanodine receptor (SPRY) or the SOCS‐box domain in SPSB1 that are both important for protein–protein interaction^17^ mediate its association with TβRII we generated two SPSB1‐SPRY domain mutants (Y129A and T160A/Y161A (TYAA)) and a mutant lacking the SOCS‐box (ΔSOCS) (Figure 2C). The SPSB1‐SPRY domain mutants have been shown to reduce the interaction between SPSB1 and its substrates.^17^ The SOCS‐box functions as a substrate recognition component of SCF‐like E3 ligases.^17^ Co‐immunoprecipitation analyses showed that SPSB1‐ΔSOCS strongly associated with TβRII in C2C12 cells, whereas the interaction between SPSB1‐Y129A and SPSB1‐TYAA with TβRII was reduced when compared with SPSB1 WT (Figure 2D). These data indicate that the SPRY‐domain of SPSB1 is involved in its interaction with TβRII in myocytes. Ubiquitination assays showed that SPSB1 increased the ubiquitination of TβRII, which was lower in SPSB1‐ΔSOCS expressing cells indicating that SPSB1‐mediates ubiquitination of TβRII via its SOCS‐box domain (Figure 2E). Cycloheximide (CHX) chase assays revealed that SPSB1 WT (Figure 2F) but not SPSB1‐ΔSOCS reduced the half‐life of endogenous TβRII in C2C12 cells (Figure S8C) indicating that SPSB1 via its SOCS‐box increases the turnover of TβRII. These data suggest that SPSB1 interacts with TβRII, mediates its ubiquitination and reduces its stability.

### SPSB1 inhibits TGF‐β signalling by targeting TGF‐β receptor II

**Figure 3:**
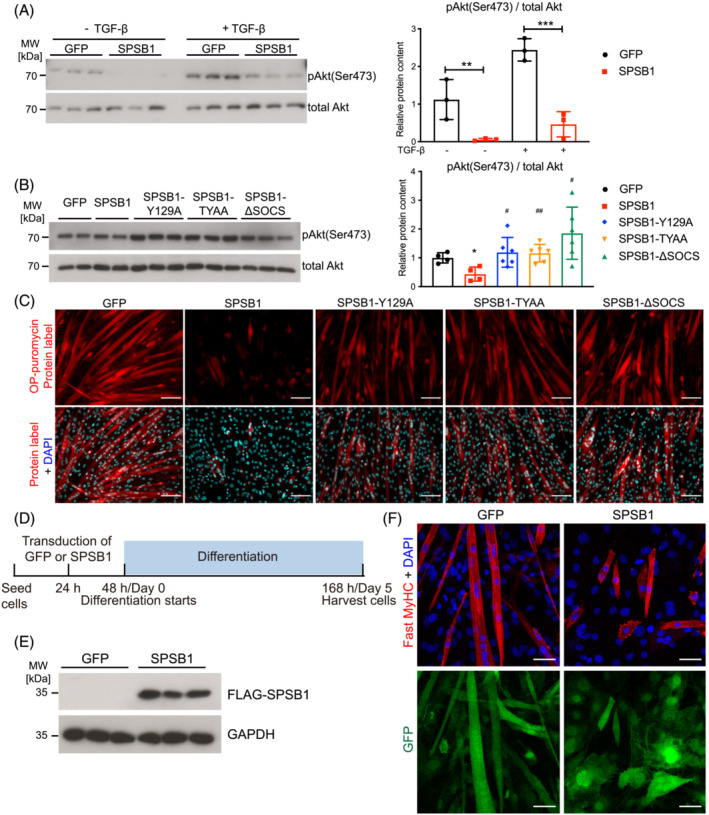
SPSB1 downregulates TGF‐β signalling by its SPRY and SOCS‐‐box domain and inhibits myogenic differentiation. (A) Five‐day‐differentiated C2C12 myotubes (MT5) transduced with GFP or SPSB1 were treated with TGF‐β (5 ng/mL) or solvent control for 5 min. Lysates were analysed by Western blot analysis with anti‐phospho Akt antibody (Ser473). Total Akt was used as control (left panel). Densitometric analysis (right panel). Data were analysed with two‐way ANOVA followed by Tukey's post‐hoc test. **P < 0.01, ***P < 0.001. (B) C2C12 cells were transduced by control GFP, SPSB1 (WT) or mutants (SPSB1‐Y129A, ‐TYAA or ‐ΔSOCS) retrovirus and differentiated for 5 days. Western blot analysis with anti‐phospho Akt antibody (Ser473) (left panel) and densitometric analysis (right panel). Total Akt was used as control. Data were analysed with one‐way ANOVA followed by Tukey's post‐hoc test. Asterisk (*) indicates significant differences between SPSB1 (wildtype or mutants as indicated) and the GFP control group, *P < 0.05, **P < 0.01, ***P < 0.001; ^#^ denotes a significant difference between indicated SPSB1 mutants and the SPSB1 wildtype group, ^#^ *P* < 0.05, ^##^ *P* < 0.01, ^###^ *P* < 0.001. (C) O‐Propargyl‐puromycin (OP‐puro) labelling of *de novo* synthesized polypeptides. Scale bar, 100 μm. (D) Experimental design. (E) Protein lysates from MT5 were analysed by Western blot with anti‐FLAG and anti‐GAPDH antibody. (F) Immunofluorescent staining with anti‐Fast MyHC antibody. Nuclei were stained with DAPI (blue). GFP (green) indicates retrovirally transduced cells. Scale bar, 50 μm.

We next investigated if SPSB1‐mediated targeting of TβRII influences canonical Smad‐dependent and non‐canonical Smad‐independent (i.e. Akt) TGF‐β signalling pathways.^21^ SPSB1 caused a nuclear‐to‐cytoplasmic translocation of Smad3 in C2C12 cells (Figure S9A,B), which was predominantly localized in the nuclei in GFP‐control cells. This was accompanied by a reduced expression of the Smad3‐target gene *Smad7* (Figure S9C). Likewise, and as expected,^21^ TGF‐β treatment increased phosphorylation of Akt (Ser473), which was attenuated by SPSB1 in C2C12 cells (Figure 3A). These data indicate that SPSB1 inhibits both canonical and non‐canonical TGF‐β signalling. Because Akt regulates protein synthesis and differentiation,^22^ which is decreased in muscle of ICUAW patients,^6^, ^10^ and because our RNA‐seq data revealed an inhibition of Akt‐signalling in muscle of septic mice,^18^ we focused on this pathway for further analyses.

### SPSB1 regulates TGF‐β signalling

We next investigated if the association of SPSB1 with TβRII is necessary for inhibition of Akt‐signalling. SPSB1 inhibited Akt (Ser473) phosphorylation in C2C12 cells, which was not observed for any of the SPSB1 mutants (Figure 3B). Smad3 was localized to the cytoplasm of SPSB1 transduced cells but remained nuclear in SPSB1‐mutant transduced cells (Figure S9A,B). Accordingly, SPSB1 but none of the SPSB1‐mutants caused a reduction in *Smad7* expression (Figure S9C). Using the O‐Propargyl‐puromycin (OP‐puro) assay we found that protein synthesis was substantially decreased by SPSB1 overexpression in C2C12 cells (Figure 3C) when compared with controls. In contrast, SPSB1 mutants had only a minor effect on protein synthesis (Figure 3C) indicating that both the SPRY‐ and the SOCS‐box domains are necessary to inhibit canonical and non‐canonical TβRII signalling and protein synthesis.

### SPSB1 overexpression inhibits myogenic differentiation

**Figure 4:**
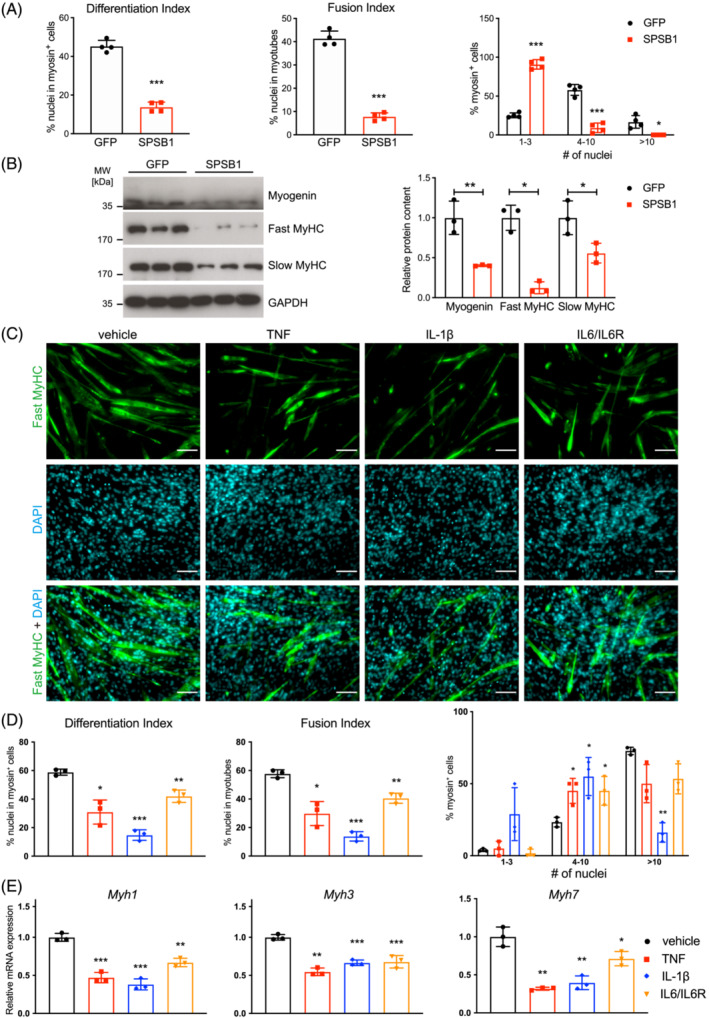
SPSB1 and proinflammatory cytokines inhibit myogenic differentiation. (A) Differentiation index, fusion index, and nuclei distribution in myosin positive (myosin^+^) cells were quantified from images in Figure 3 panel (F). (B) Western blot analysis of lysates from GFP and SPSB1 transduced cells that were differentiated for 5 days with indicated antibodies. GAPDH was used as loading control. Densitometric analysis is shown in the right panel. Data in panel (A; Differentiation and fusion index) and (B) were analysed with two‐tailed Student's *t*‐test; data in panel A (myosin^+^ cells) were analysed with two‐way ANOVA followed by Tukey post hoc test; asterisk (*) indicates a significant difference of SPSB1 (WT) or mutants compared with GFP group, *P < 0.05, **P < 0.01, ***P < 0.001; ^#^ indicates a significant difference of SPSB1 mutants compared with SPSB1 (WT) group, ^#^ *P* < 0.05, ^##^ *P* < 0.01, ^###^ *P* < 0.001. *N* = 3 biologically independent experiments; data are presented as mean ± standard deviation. (C) Immunofluorescent staining of 5 days differentiated C2C12 cells treated with TNF (10 ng/mL), IL‐1β (10 ng/mL) or IL6/IL6R (100 ng/mL), as indicated, every 24 h throughout differentiation, with anti‐fast MyHC antibody. Nuclei were stained with DAPI (blue). Scale bar, 100 μm. (D) Differentiation index, fusion index, and nuclei distribution in myosin^+^‐cells were quantified from images in panel (C). (E) qRT‐PCR analysis of *Myh1*, *Myh3*, and *Myh7*. mRNA expression was normalized to *Gapdh*. Data in panel (D; Differentiation and fusion index) were analysed with two‐tailed Student's *t*‐test; data in panel (D; myosin^+^‐cells) and (E) were analysed with two‐way ANOVA followed by Tukey post hoc test; *P < 0.05, **P < 0.01, ***P < 0.001. *N* = 3 biologically independent experiments; data are presented as mean ± standard deviation.

We consistently observed that SPSB1 overexpression inhibited myotube formation (Figure 3C,F). To investigate this phenotype, we retrovirally transduced SPSB1‐IRES‐GFP or IRES‐GFP‐control into C2C12 myoblasts and assessed myogenic differentiation (workflow in Figure 3D,E, Figure S10A), which was indeed inhibited by SPSB1 (Figure S10B). Immunocytochemistry revealed that SPSB1 overexpressing cells contained less fast myosin heavy chain protein (MyHC), a marker of terminal differentiation, were thinner and shorter, and comprised fewer nuclei when compared with GFP control cells on differentiation day 5 (Figure 3F). Quantification of differentiation and fusion indices, and the number of nuclei per myosin^+^‐cell showed that SPSB1 transduced cells did not differentiate and remained mono‐nucleated (Figure 4A,B). SPSB1 overexpression caused a reduction of the differentiation markers Myogenin/*Myog*, Myomaker/*Mymk* and Myomerger/*Mymx* and MyHC/*Myh* throughout differentiation (Figure S10C). Accordingly, Myogenin, and fast and slow MyHC were reduced in SPSB1 overexpressing cells (Figure 4B). These data show that SPSB1 inhibits both myoblast fusion and myogenic differentiation preventing myotube formation.

These effects of SPSB1 could be involved in the impaired regenerative capacity found in muscle of ICU patients^8^, ^9^ and septic mice.^10^ During regeneration muscle residing stem cells, called satellite cells, provide myogenic cells that proliferate, differentiate, fuse, and form new functional myofibers.^11^ Therefore, if SPSB1 is involved in myogenic differentiation it should be contained in satellite cells. To test this hypothesis, we analysed single‐cell RNAseq data from the *Tabula Muris Consortium* ^23^ and found *Spsb1* but not *Spsb2*, *Spsb3*, or *Spsb4* to be enriched in satellite and mesenchymal stem cells in murine limb muscles (Figure S11A,B). Because proinflammatory cytokines increased *Spsb1* expression, we reasoned that they would also inhibit myogenic differentiation. Indeed, when we treated C2C12 myoblasts with TNF, IL‐1β, and IL‐6/IL‐6R, respectively, for 5 days myogenic differentiation was greatly perturbed (Figure 4C,D), which was also reflected by a decreased expression of late differentiation markers (*Myh1*, *Myh3*, *Myh7*) (Figure 4E).

**Figure 5:**
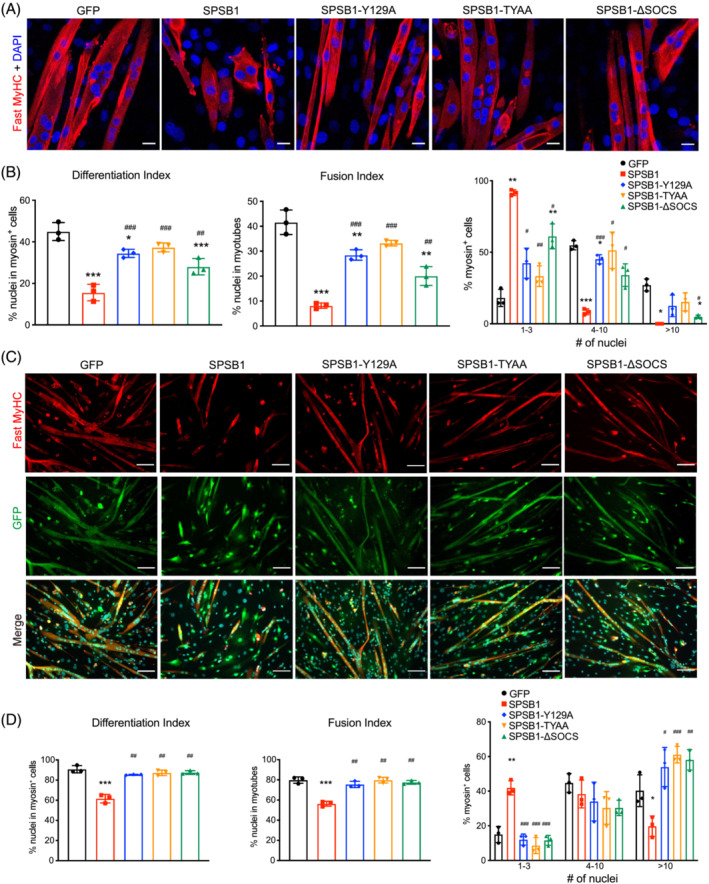
SPSB1 mediated inhibition of myogenic differentiation depends on its SPRY and SOCS‐‐box domain. (A, B) C2C12 myoblasts were transduced by control GFP, SPSB1 (WT) or mutant (SPSB1‐Y129A, ‐TYAA or ‐ΔSOCS) containing retrovirus and differentiated for 5 days. (A) Immunofluorescent staining with anti‐fast MyHC antibody. Nuclei were stained with DAPI (blue). Scale bar, 20 μm. (B) Differentiation index, Fusion index, and Nuclei distribution in each myosin^+^ cell were quantified from images in panel (A). (C, D) Primary myoblasts were transduced by control GFP, SPSB1 (WT) or mutant (SPSB1‐Y129A, ‐TYAA or ‐ΔSOCS) containing retrovirus and differentiated for 5 days. (C) Immunofluorescent staining with anti‐fast MyHC antibody (red). GFP (green) indicates retrovirally transduced cells. Scale bar, 100 μm. (D) Differentiation index, Fusion index, and Nuclei distribution in each myosin^+^ cell were quantified from images in panel (C). Data in panels (B and C; Differentiation and Fusion index), were analysed with two‐tailed Student's *t*‐test; data in panels (B) and (H) (Nuclei distribution in myosin^+^ cells) were analysed with two‐way ANOVA followed by Tukey's post‐hoc test; asterisk (*) indicates significant differences between SPSB1 (wildtype or mutants as indicated) and the GFP control group, *P < 0.05, **P < 0.01, ***P < 0.001; denotes a significant difference between indicated SPSB1 mutants and the SPSB1 wildtype group, ^#^ *P* < 0.05, ^##^ *P* < 0.01, ^###^ *P* < 0.001. *N* = 3 biologically independent experiments; data are presented as mean ± standard deviation.

In contrast to SPSB1 WT, overexpression of the SPSB1‐SPRY and SPSB1‐SOCS box mutants had only a minor effect of myogenesis (Figure 5A,B), which was supported by qRT‐PCR (Figure S12A) and Western blot (Figure S12B,C) analyses for early (*Myog*, *Mymk*, *Mymx*) and late (*Myh1*, *Myh3*, *Myh7*) myogenic markers. SPSB1 overexpression also inhibited myogenic differentiation in commercial mouse skeletal muscle myoblasts, which was not observed for any of the SPSB1‐mutants (Figure 5C,D). Our data suggest that SPSB1 attenuates myogenic differentiation by inhibition of TβRII signalling and that this effect depends on its SPRY‐ and SOCS‐box‐domains.

### TGF‐β signalling is essential for myogenesis

To further investigate if TGF‐β via TβRII plays a role in myogenesis, we examined the amount of TβRII protein and *Tgfb1* gene expression in differentiating C2C12 cells and found them to be increased (Figure S13A,B). Immunohistochemistry revealed that TβRII was enriched in myosin^+^‐myotubes (Figure S13C). To explore if TβRII is essential for myogenesis, we used the TβRII‐specific inhibitor *inducer of TβRII receptor degradation‐1* (ITD‐1).^24^ ITD‐1 caused a dose‐dependent reduction of TβRII (Figure S14A), a decreased Akt (Ser473) phosphorylation (Figure S14B) and a reduced protein synthesis (Figure S14C) in C2C12 cells when compared with vehicle‐treated cells. ITD‐1 impaired myogenesis as shown by shorter myosin^+^‐myotubes with fewer nuclei (Figure S14D), inhibited myoblast fusion and differentiation (Figure S14E) and decreased the expression of early (*Myog*, *Mymx*) and late (*Myh3*) myogenic markers (Figure S13C,D) and Myogenin protein content in differentiating C2C12 cells (Figure S14FA). The amount of slow‐twitch but not fast‐twitch MyHC was reduced by ITD‐1 (Figure S14F). ITD‐1 also caused an increase in some myogenic markers (*Myh1*, *Myh7*) (Figure S13D). These data suggest that TGF‐β/TβRII‐Akt‐signalling plays a role in protein synthesis and myogenesis.

### Akt restores myogenesis in SPSB1 overexpressing cells

**Figure 6:**
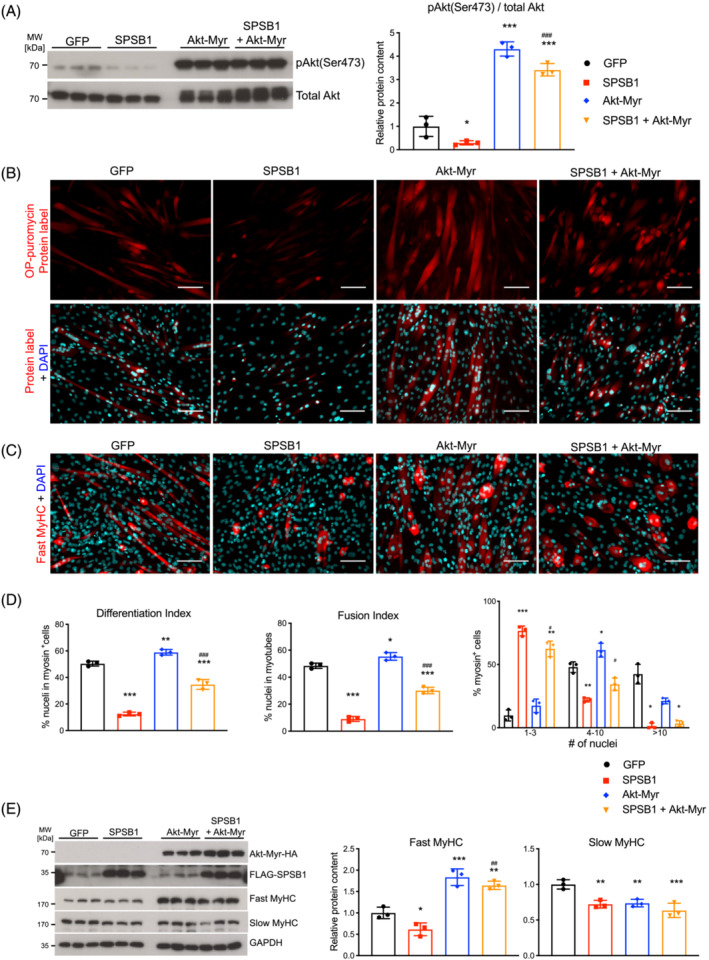
Expression of Akt restores myogenesis in SPSB1 overexpressing cells. Cells were transduced by control GFP, SPSB1, Akt‐Myr, respectively, or co‐transduced by Akt‐Myr and SPSB1 retrovirus and differentiated for 5 days. (A) Western blot analysis of anti‐phospho Akt antibody (Ser473). Total Akt was used as control. Densitometric analysis is displayed in the right panel. (B) Cells described above were incubated with OP‐puro labelling for 1 h. Red fluorescence (upper panel) corresponds to *de novo* synthesized polypeptides. (C) Immunofluorescent staining of above cells with anti‐Fast MyHC as primary antibody and Alexa Fluor 555 conjugated secondary antibody (red). Scale bar, 100 μm. (D) Differentiation index, Fusion index, and Distribution of nuclei in myosin positive (myosin^+^) cells were quantified from images in panel (C). (E) Western blot was performed using lysates from above cells with anti‐Fast MyHC and anti‐Slow MyHC antibody. Overexpressed Akt‐Myr and SPSB1 were detected by anti‐HA and anti‐FLAG antibody, respectively. GAPDH was used as loading control. Densitometric analysis of Western blot signals are displayed (right panel). Data in panels (A), (D; differentiation and fusion index) and (E) were analysed with one‐way ANOVA followed by Tukey's post‐hoc test; data in panel (D; myosin^+^ cells) were analysed with two‐way ANOVA followed by Tukey's post‐hoc test. Asterisk (*) indicates a significant difference between SPSB1‐, Akt‐Myr‐ or SPSB1 and Akt‐Myr‐treated groups compared with GFP control treated cells, *P < 0.05, **P < 0.01, ***P < 0.001; ^#^ indicates a significant difference between SPSB1‐ and SPSB1 + Akt‐Myr‐treated cells, ^#^ *P* < 0.05, ^##^ *P* < 0.01, ^###^ *P* < 0.001. *N* = 3 biologically independent experiments; data are presented as mean ± standard deviation.

As SPSB1‐mediated inhibition of TβRII‐Akt signalling inhibited myogenesis, we tested if co‐expression of myristylated, constitutively active Akt (Akt‐Myr) restores myogenesis in SPSB1 overexpressing cells. Transduction of Akt‐Myr resulted in an increased Akt (Ser473) phosphorylation (Figure 6A), an increased protein synthesis and an elevation of fast‐twitch MyHC in C2C12 cells (Figure 6B,C). Akt‐Myr reverted the inhibitory effects of SPSB1 on protein synthesis (Figure 6B), myogenic differentiation, myoblast fusion (Figure 6D) and MyHC content (Figure 6E) as well as myogenic factors (*Myog*, *Mymk*, *Mymx*) (Figure S15A) and terminal differentiation markers (*Myh1*, *Myh3*) in C2C12 cells (Figure S15B). However, *Myh7* expression (Figure S15B) and slow‐twitch MyHC (Figure 6E) remained unaffected by Akt‐Myr. Akt‐Myr also reverted SPSB1‐mediated inhibition of protein synthesis, myogenic differentiation, and myoblast fusion (Figures S16A,B and S17A) in self‐isolated and commercial primary myoblasts. Together, these data show that Akt‐Myr rescues the inhibitory effects of SPSB1 on protein synthesis, myoblast fusion and myogenic differentiation.

### Myogenin facilitates differentiation in SPSB1 transduced cells

**Figure 7:**
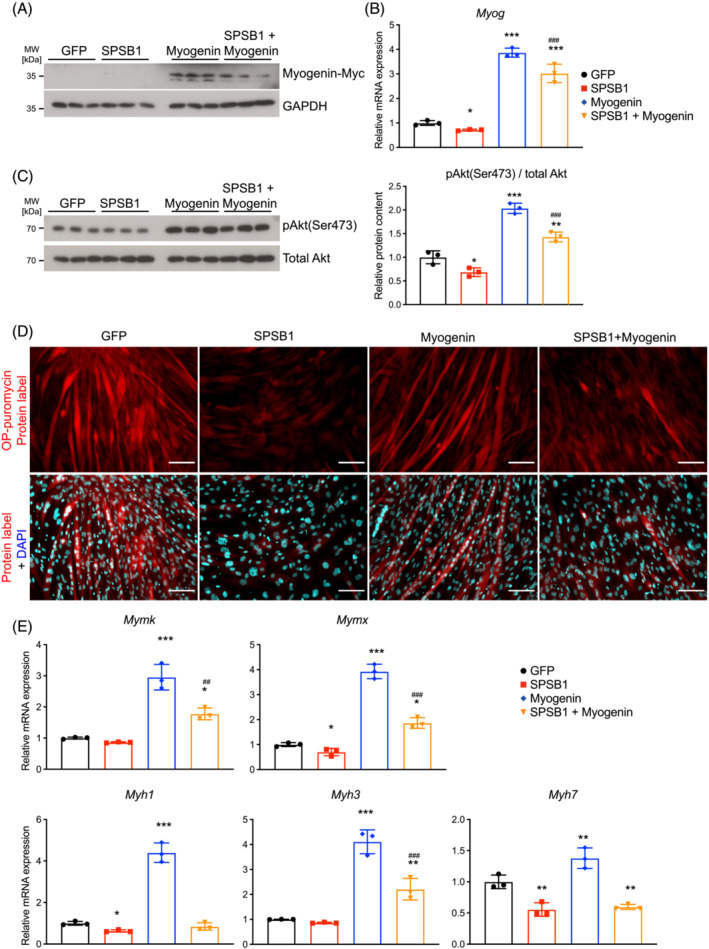
Myogenin restores Akt signalling and protein synthesis in SPSB1 overexpressing cells. C2C12 cells were transduced by control GFP, SPSB1, Myogenin, respectively, or co‐transduced by Myogenin and SPSB1 retrovirus and differentiated for 5 days. (A) Western blot with anti‐Myc antibody. GAPDH was used as loading control. (B) qRT‐PCR analysis of *Myog* mRNA expression normalized to *Gapdh*. (C) Western blot analysis with anti‐phospho Akt antibody (Ser473). Total Akt was used as control. Densitometric analysis is shown in the right panel. (D) O‐Propargyl‐puromycin (OP‐puro) assay: Red fluorescence (upper panel) corresponds to *de novo* synthesized polypeptides. (E) qRT‐PCR analysis of *Mymk*, *Mymx*, *Myh1*, *Myh3*, and *Myh7* from described cells. mRNA expression was normalized to *Gapdh*. Data were analysed with one‐way ANOVA followed by Tukey's post‐hoc test. Asterisk (*) indicates a significant difference between SPSB1‐, Myogenin‐ or SPSB1 + Myogenin‐ and GFP control groups, *P < 0.05, **P < 0.01, ***P < 0.001; ^#^ indicates a significant difference between SPSB1‐ and SPSB1 + Myogenin‐treated cells, ^#^ *P* < 0.05, ^##^ *P* < 0.01, ^###^ *P* < 0.001. *N* = 3 biologically independent experiments; data are presented as mean ± standard deviation.

**Figure 8:**
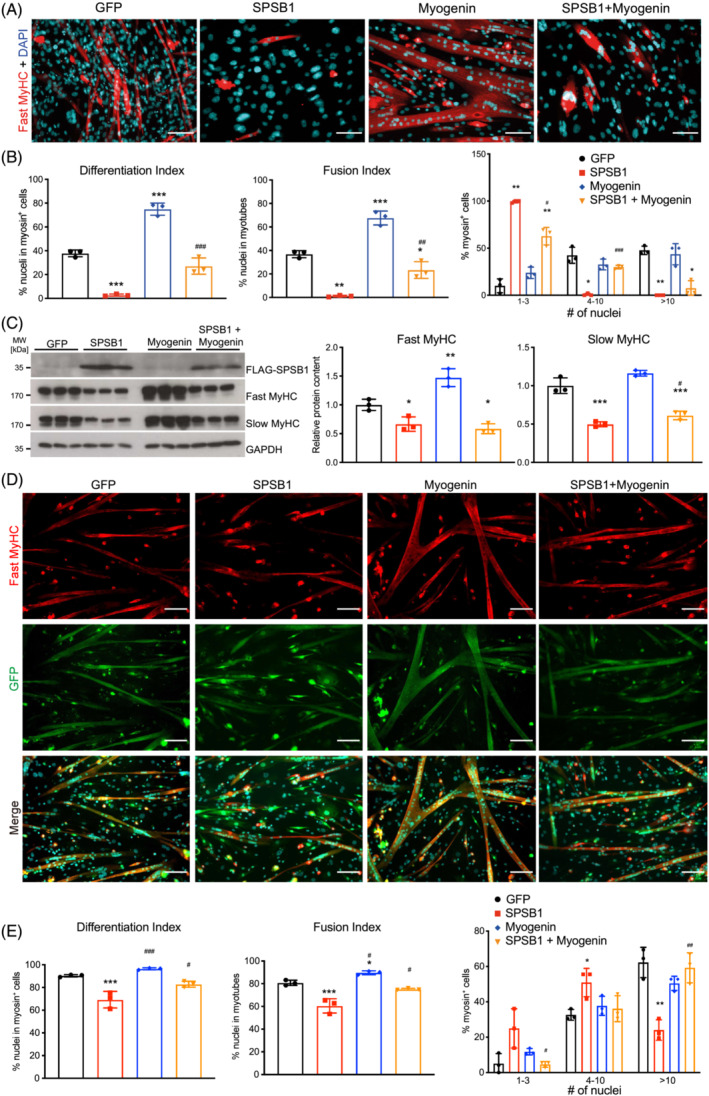
Myogenin restores myogenesis in SPSB1 overexpressing cells. (A–C) C2C12 cells were transduced by control GFP, SPSB1, Myogenin, respectively, or co‐transduced by Myogenin and SPSB1 retrovirus and differentiated for 5 days. (A) Immunofluorescent staining of above cells with anti‐Fast MyHC as primary antibody and Alexa Fluor 555 conjugated secondary antibody (red). Scale bar, 100 μm. (B) Differentiation index, Fusion index, and Distribution of nuclei in myosin positive (myosin^+^) cells were quantified from images in panel (A). (C) Western blot was performed using lysates from above cells with anti‐Fast MyHC and anti‐Slow MyHC antibody. Overexpressed SPSB1 was detected by anti‐FLAG antibody. GAPDH was used as loading control. Densitometric analysis of Western blot signals are displayed (right panel). Data in panels (B; Differentiation and Fusion index) and (C) were analysed with one‐way ANOVA followed by Tukey's post‐hoc test; data in panel (B; myosin^+^ cells) were analysed with two‐way ANOVA followed by Tukey's post‐hoc test. Asterisk (*) indicates a significant difference between SPSB1‐, Myogenin‐ or SPSB1 + Myogenin‐ and GFP control groups, *P < 0.05, **P < 0.01, ***P < 0.001; ^#^ indicates a significant difference between SPSB1‐ and SPSB1 + Myogenin‐treated cells, ^#^ *P* < 0.05, ^##^ *P* < 0.01, ^###^ *P* < 0.001. *N* = 3 biologically independent experiments; data are presented as mean ± standard deviation. (D, E) Primary myoblasts were transduced by control GFP, SPSB1, Myogenin, respectively, or co‐transduced by Myogenin and SPSB1 retrovirus and differentiated for 5 days. (D) Immunofluorescent staining of above cells with anti‐Fast MyHC as primary antibody and Alexa Fluor 555 conjugated secondary antibody (red). GFP (green) indicates retrovirally transduced cells. Scale bar, 100 μm. (E) Differentiation index, Fusion index, and Nuclei distribution in each myosin^+^ cell were quantified from images in panel (D). Data in panel (E; Differentiation and Fusion index) were analysed with one‐way ANOVA followed by Tukey's post‐hoc test; data in panel (E; myosin^+^ cells) were analysed with two‐way ANOVA followed by Tukey's post‐hoc test. Asterisk (*) indicates a significant difference between SPSB1‐, Myogenin‐ or SPSB1 + Myogenin‐ and GFP control groups, *P < 0.05, **P < 0.01, ***P < 0.001; ^#^ indicates a significant difference between SPSB1‐ and SPSB1 + Myogenin‐treated cells, ^#^ *P* < 0.05, ^##^ *P* < 0.01, ^###^ *P* < 0.001. *N* = 3 biologically independent experiments; data are presented as mean ± standard deviation.

Because Akt increases *Myog* expression,^25^ a key factor for differentiation,^26^ we tested if SPSB1‐mediated inhibition of myogenesis involves Myogenin. Indeed, SPSB1 reduced Myogenin mRNA expression and protein content in differentiating C2C12 cells (Figure 4B, Figure S10C). Restoration of Myogenin expression in SPSB1‐transduced myocytes increased Akt (Ser473) phosphorylation (Figure 7A–C), enhanced protein synthesis (Figure 7D) and elevated the expression of myogenic factors (*Mymk*, *Mymx*) and terminal differentiation markers (*Myh3*) but not *Myh1* and *Myh7* (Figure 7E). These effects were accompanied by an increase in differentiation and fusion indices (Figure 8A,B). Co‐expression of Myogenin and SPSB1 improved myogenic differentiation and increased fast‐ and slow‐twitch MyHC proteins (Figure 8C). Myogenin also reverted SPSB1‐mediated inhibition of protein synthesis (Figure S17B), myogenic differentiation, and myoblast fusion (Figure 8D,E) in self‐isolated and commercial primary myoblasts. In summary, these data show that Myogenin reverses the inhibitory effects of SPSB1 on protein synthesis, myoblast fusion and myogenic differentiation.

### Myocyte‐specific knockdown of Spsb1 reduces sepsis‐induced muscle atrophy in mice

To test the hypothesis that knockdown of *Spsb1* prevents muscle atrophy in septic mice, we generated short hairpin RNA (shRNA) to knock down *Spsb1* directly in skeletal myocytes. We injected 1*10^12^ vg of AAV9 Spsb1_shRNA or AAV9‐control_shRNA into the tail vein of male 8‐week‐old B6(C)/Rj‐Tyr^c/c^ mice. Six weeks later, we performed CLP‐ or sham‐surgery for 96 h (Experimental design in Figure S18A). Spsb1_shRNA blunted CLP‐induced expression of *Spsb1* in TA and GP (Figure S18B). Sepsis caused a reduction in body (control_shRNA: −23%, *P* < 0.001; Spsb1_shRNA: −9%, *P* = 0.3), TA (−26%, *P* < 0.01) and GP (−21%, *P* < 0.01) weights, which was reduced by *Spsb1* knockdown (Figure S18C). Quantification of haematoxylin and eosin stained histological cross‐sections showed a reduction in MCSA of septic control_shRNA treated mice that was attenuated in TA of septic Spsb1_shRNA mice (control_shRNA: −25.4%, Spsb1_shRNA: −9.2%; *P* < 0.001; Figure S18D). To investigate any effects on protein degradation, we quantified the expression of the atrophy genes *Trim63*/MuRF1 and *Fbxo32/*Atrogin‐1. Spsb1 knockdown attenuated sepsis‐induced increases in *Trim63* and *Fbxo32* expression (Figure S18E,F). These data indicate that inhibition of SPSB1 reduces inflammation‐induced muscle atrophy in mice.

## Discussion

We identified SPSB1 as a novel regulator of the TβRII‐Akt‐Myogenin pathway in skeletal muscle and myocytes. SPSB1 associates with, ubiquitinates and reduces the stability of TβRII and inhibits TβRII‐signalling resulting in reduced protein synthesis and attenuated myogenic differentiation. As SPSB1 is significantly induced in skeletal muscle of critically ill patients and in septic mice it may contribute to the observed inhibition of myogenic differentiation in muscle of septic patients and mice. In line with previously published work on non‐myocytes,^17^ we proved that SPSB1 and TβRII physically interact and colocalize in myocytes, which facilitates ubiquitination of TβRII and reduces its stability. This in turn inhibited both canonical and non‐canonical TβRII‐signalling and negatively affected myocyte homeostasis. SPSB1 overexpression inhibited protein synthesis, myoblast fusion and myogenic differentiation, which lead to a decrease in early and late differentiation markers. We uncovered that the SPRY‐ and the SOCS‐box domain of SPSB1, are involved in its interaction with TβRII and the regulation of Akt and Smad3 signalling. These data indicate that the SPRY‐domain and the SOCS‐box of SPSB1 are important for its inhibitory function on TβRII‐induced myogenesis (Figure S19).

Myogenesis is a highly ordered process controlled by multiple factors, including MyoD and Myogenin.^27^, ^28^ Besides, Myomaker/*Mymk* and Myomerger/*Mymx* that mediate myoblast fusion terminal differentiation markers especially MyHC are coordinately expressed.^12^, ^13^, ^14^ Our findings indicate that SPSB1 inhibits myogenesis by suppression of the TGF‐β/TβRII‐Akt‐Myogenin axis. In line with previous work,^25^ we showed that Akt induces *Myog* expression and enhances myoblast fusion and myogenic differentiation. Furthermore, Myogenin increases the expression of *Mymk* and *Mymx*,^29^ which was also observed in our study. In contrast, SPSB1 inhibited myogenic differentiation, which was accompanied by a decreased expression of all myogenic factors. The SPSB1‐phenotype was restored when Akt or Myogenin were co‐expressed with SPSB1 in primary and immortalized myoblasts. Our data, together with previous work, thus indicate that the TGF‐β/TβRII‐Akt‐Myogenin axis regulates myogenesis.

The strong increase in contractile and surface proteins during myogenesis suggests that protein synthesis is required for this process.^12^, ^13^ This is supported by the observation that inhibition of protein synthesis leads to a decrease in of C2C12 myoblast fusion.^30^ Here, we show that inactivation of TβRII by SPSB1 and ITD‐1 reduces protein synthesis as well as myogenesis, further indicating that both processes are interconnected.

TGF‐β family members and their downstream effectors Smad2 and Smad3 were reported to inhibit myogenesis.^31^, ^32^ In contrast, other groups showed that Smad3 and Smad7 are essential for myogenesis.^33^, ^34^ However, only few groups examined the function of TGF‐β receptors on myotube formation. Our data provide evidence that TβRII promotes myogenesis. Specifically, TβRII expression was increased during differentiation and was enriched in myosin^+^‐cells. Myoblasts treated with the TβRII inhibitor ITD‐1 failed to form myotubes, which is in agreement with the observation that myoblasts expressing a dominant negative TβRII did not differentiate.^35^ We also showed that TGF‐β/TβRII‐Akt‐Myogenin is essential for myogenesis. Our observation that ITD‐1 inhibited both myoblast fusion and myogenic differentiation is in contrast to a recent report that showed that ITD‐1 promotes fusion without affecting differentiation in primary myoblasts.^36^ In contrast to our study, the authors did not observe an upregulation of TβRII during differentiation indicative for methodological differences between our respective analyses. In addition, our observation that ITD‐1 treatment caused an increase of fusion‐related factors (*Mymk*, *Mymx*) during early, but not late differentiation suggests that TGF‐β/TβRII signalling has distinct functions throughout differentiation. This hypothesis is supported by findings of Willems et al. who reported that ITD‐1 abolished cardiogenesis in mouse embryonic stem cells when added between days 1–3 of differentiation but promoted it from days 3–5.^24^ Moreover, the different downstream effectors of TGF‐β also seem to differ in their effects on differentiation. For example, Melendez et al. showed that TGF‐β, via Smad2/3, inhibited myoblast fusion but not differentiation in chicken embryos.^37^ Our study shows that non‐canonical TGF‐β signalling via Akt‐Myogenin promotes both myoblast fusion and differentiation. Collectively, these findings suggest that the effects of TGF‐β/TβRII‐signalling on myocyte biology vary depending on the models used.

SPSB1 shares 50% and 75% amino acid sequence identity with SPSB2 and SPSB4, respectively,^19^ whereas SPSB3 shares the least amino acid sequence identity with other SPSB‐family members.^19^ SPSB proteins have some overlapping but also distinct targets and therefore functions. For example, SPSB1 and SPSB4, but not SPSB3 interact with human prostate apoptosis response protein‐4 (hPar‐4). SPSB2 also interacts with Par‐4, but not as strong.^38^ Other SPSB proteins may therefore also play a role in TGF‐β/TβRII‐signalling and myogenesis. However, first, only SPSB1 was shown to be involved in TGF‐β‐signalling^17^ and second, only SPSB1 was highly expressed in satellite cells indicating a specific role in this cell type. Third, unlike *Spsb1* the other SPSB‐family members were either only regulated in some muscles, at different time points or not regulated during sepsis in mice. Interestingly, *Spsb3* expression was increased in all muscles but only 96 h after CLP surgery in mice. By contrast, *SPSB1*, *SPSB2*, and *SPSB3* were increased in muscle of ICUAW patients, indicating that also SPSB2 and SPSB3 are also involved in inflammation‐induced muscle pathologies. However, further studies are needed to investigate their specific functions in muscle.

The proposed model that inflammatory cytokines induce SPSB1 to block TGF‐β signalling and myogenesis could also be interpreted as a lifesaving mechanism during the early stages of sepsis, which may result in failed regeneration during the later disease course. Inflammatory cytokines and sepsis lead to a disturbed protein homeostasis with a decreased synthesis and an increased degradation.^4^, ^5^, ^6^, ^18^ Others and we hypothesize that muscle serves as a reservoir for proteins that are used as fuel to assure survival during critical illness. However, once the organism survives sepsis breakdown of muscle tissue can lead to weakness. As protein synthesis is energy demanding its inhibition could be beneficial during sepsis. In this regard, the cytokine‐mediated increase in SPSB1 could inhibit TGF‐β‐induced protein synthesis, satellite cell proliferation and muscle repair to prioritize defence mechanisms in sepsis. However, the same mechanisms could lead to failed regeneration in ICUAW. Because, TGF‐β via TβRII also increases the expression of SPSB1^17^ that in turn inhibits TβRII signalling this regulation could facilitate a negative feed‐back loop. Finally, TGF‐β plays a crucial role in inflammation and immune responses and has both pro‐ and anti‐inflammatory properties.^39^ TGF‐β can promote inflammation by stimulating the recruitment and activation of immune cells and increase the expression of pro‐inflammatory cytokines, such as TNF and IL‐6, and activate the NF‐κB pathway, which all cause muscle wasting.^4^, ^5^, ^18^ An increased expression of SPSB1 could therefore be beneficial to limit TGF‐β‐induced inflammation in sepsis. However, if SPSB1 also inhibits anti‐inflammatory TGF‐β‐functions in muscle during sepsis warrants further investigation.

The inhibition of TGF‐β/TβRII‐Akt‐Myogenin signalling by SPSB1 may shed light on the pathogenesis of the perturbed regenerative capacity observed in muscle of critically ill patients with sepsis (Figure S19). Protein synthesis, which is critical for maintenance of muscle size and function, is reduced in muscle of critically ill patients.^6^ Our data implicate that TβRII‐mediated Akt activation plays a role in maintaining homeostasis in muscle and myocytes in men and mice and that protein synthesis is required for myogenesis. We show that SPSB1 overexpression inhibits protein synthesis and myogenic differentiation, and these effects were rescued by Akt and Myogenin. Because a reduced number and malfunction of satellite cells as well as an impaired muscle regeneration have been reported in ICUAW patients and septic mice,^8^, ^9^, ^10^ we propose that SPSB1‐mediated inhibition of TβRII‐Akt‐Myogenin signalling and myogenesis contributes to a disturbed muscle homeostasis that occurs during inflammation.

## Limitations

In our short‐term sepsis mouse model, we observed a 20‐ to 34‐fold increase in muscular *Spsb1* expression 24 and 96 h after surgery, respectively. An increased muscular *SPSB1* expression was confirmed in muscle of ICUAW patients; however, only twofold. This discrepancy is possibly due to differences in species and muscle tissue investigated, the time point of analyses, and the underlying causes of critical illness as well as its treatment. Additionally, data from patients and from a well‐defined and standardized mouse model in which only male C57BL/6 J mice of the same age were used may differ due to the experimental design and the heterogeneity of the study subjects. Our mechanistical data on the role of SPSB1 in myogenic differentiation suggest the TβRII‐Akt‐Myogenin pathway contributes to defective muscle regeneration and myopathy observed in ICUAW patients. However, if SPSB1 is involved in the pathomechanisms underlying the up to 5 years persistent failure to regenerate cannot be answered by our work and warrants further investigations.

## Conflict of interest

The authors declare no conflict of interest.

## Supporting information


**Table S1.** Primers for generation of cDNA expression plasmids.
**Table S2.** Primers for generation of retroviral expression plasmid.
**Table S3.** Primers of site‐direct mutagenesis using SPSB1‐retroviral plasmid as template.
**Table S4.** Primers for quantitative real‐time PCR.
**Figure S1.** Quantitative RT‐PCR (qRT‐PCR) analysis of *Spsb1*, *Spsb2*, *Spsb3*, and *Spsb4* from the tibialis anterior (TA), gastrocnemius plantaris (GP), soleus (Soleus), and extensor digitorum longus (EDL) muscle of 12‐week‐old male C57BL/6 J mice subjected to cecal ligation and puncture (CLP, *n* = 5, 24 h; n = 5–9, 96 h) or sham surgery (sham, *n* = 3, 24 h; n = 3–5, 96 h). Data in were analysed with two‐way ANOVA followed by Tukey's post‐hoc test. *p < 0.05, **p < 0.01, ***p < 0.001.
**Figure S2.** Immunofluorescent staining of mouse TA muscle using anti‐SPSB1 (red), anti‐MyHC‐2A (green), anti‐MyHC‐2B (cyan), and anti‐Laminin (white) antibodies. Stars indicate enrichment of SPSB1 and in MyHC‐2A containing cells. Arrowheads reveal TβRII was absent on cytoplasmic membrane. Scale bar, 50 μm. Data in were analysed with two‐way ANOVA followed by Tukey's post‐hoc test. *p < 0.05, **p < 0.01, ***p < 0.001.
**Figure S3.** qRT‐PCR of *SPSB2* (A), *SPSB3* (B), and *SPSB4* (C) from the *vastus lateralis* muscle of patients with intensive care unit‐acquired weakness (ICUAW, *n* = 7) compared to healthy subjects (controls, *n* = 12). mRNA expression was normalized to *GAPDH*. Data were analysed with two‐tailed Student's *t*‐test. ***p < 0.001.
**Figure S4.** (A) qRT‐PCR of *Spsb1* from five‐day‐differentiated C2C12 myotubes (MT5) that were treated with TNF (10 ng/ml), IL‐1β (10 ng/ml) or IL6/IL6R (100 ng/ml) for indicated time points. (B) qRT‐PCR analysis of *Spsb1* from five‐day‐differentiated C2C12 myotubes treated with SAA1 (10 ng/ml) or LPS (1 μg/mL) for 72 h. (C) qRT‐PCR analysis of *Spsb1* from C2C12 myotubes that were treated with TGFβ (0.2 or 5 ng/ml) for 24 h and 72 h, as indicated.
**Figure S5.** Immunofluorescent staining of TA muscle from sham or CLP operated mice after 96 h using anti‐TβRII (red) and anti‐MyHC‐2A (green) antibody. Nuclei were stained with DAPI (blue). Arrowheads indicate reduction of TβRII at the cytoplasmic membrane. Scale bar, 100 μm.
**Figure S6.** Heat map of significantly regulated genes (*p* < 0.05) contained in Gene Ontology (GO) term analysis (Biological process) ‘cellular response to transforming growth factor beta stimulus’ (GO:0071560), where they were significantly enriched (*p* = 8.84E‐05, FDR 0.002), in tibialis anterior muscle of septic wildtype mice 24 h and 96 h after surgery (*n* = 3 for each condition).
**Figure S7.** (A) Heat map of significantly regulated genes (*p* < 0.05) contained in Kyoto Encyclopedia of Genes and Genomes (KEGG)‐pathway ‘Transforming growth factor‐beta signalling pathway’ (mmu04350), where they were significantly enriched (*p* = 5.4E‐04, FDR 0.0038), in tibialis anterior muscle of septic wildtype mice 24 h and 96 h after surgery (*n* = 3 for each condition). (B) Position of significantly regulated genes in KEGG‐pathway mmu04350. Regulated genes are shown in red.
**Figure S8.** (A) Subcellular distribution of SPSB1‐Myc, FLAG‐TβRII or FLAG‐TβRII‐ΔEx2 separately transfected C2C12 cells as detected by immunofluorescence using anti‐Myc antibody together with A555‐coupled secondary antibody (red) or anti‐FLAG antibody together with A488‐coupled secondary antibody (green). (B) Subcellular distribution and co‐localization of endogenous TβRII and SPSB1‐Myc in transfected C2C12 cells. Anti‐TβRII together with A488‐coupled secondary antibody (green) and anti‐Myc antibody together with A555‐coupled secondary antibody (red) were used. Nuclei were stained with DAPI (blue). Scale bar, 20 μm. (C) Cells were transduced with a retrovirus encoding GFP (control), SPSB1 or SPSB1‐ΔSOCS for 48 h and then treated with cycloheximide (CHX, 50 μg/ml) as indicated. Western blot analysis of cell lysates. Anti‐FLAG antibody shows over‐expressed FLAG‐SPSB1. GAPDH was used as loading control. Densitometric analysis.
**Figure S9.** C2C12 cells were transduced by control GFP, SPSB1 (WT) or mutants (SPSB1‐Y129A, ‐TYAA or ‐ΔSOCS) retrovirus and differentiated for 5 days. (A) Immunofluorescence of Smad3 (red) and DAPI (blue). Arrowheads shows co‐localization of Smad3 and nuclei in GFP and SPSB1‐mutant transduced cells. Scale bar, 20 μm. (B) Quantification of the percentage of Smad3 positive (Smad3+) nuclei. (C) qRT‐PCR analysis of TGF‐β‐Smad3 responsive gene *Smad7*. Data were analysed with one‐way ANOVA followed by Tukey's post‐hoc test. * indicates significant differences between SPSB1 (wildtype or mutants as indicated) and the GFP control group, *p < 0.05, **p < 0.01, ***p < 0.001; # denotes a significant difference between indicated SPSB1 mutants and the SPSB1 wildtype group, #*p* < 0.05, ##*p* < 0.01, ###*p* < 0.001; *n* = 3 biologically independent experiments; data are presented as Mean ± standard deviation.
**Figure S10.** C2C12 cells were transduced with control GFP or SPSB1 retrovirus and differentiated for 1, 3 or 5 days. (A) qRT‐PCR analysis of *Spsb1* from C2C12 cells at indicated timepoints. (B) Direct imaging of transduced C2C12 cells at indicated timepoints. GFP signals denote transduced cells. Scale bar, 100 μm. (C) qRT‐PCR analysis of *Myog*, *Mymk*, *Mymx*, *Myh1*, *Myh3,* and *Myh7*. mRNA expression was normalized to *Gapdh*. Data were analysed with two‐way ANOVA followed by Tukey's post‐hoc test. *p < 0.05, **p < 0.01, ***p < 0.001. *n* = 3 biologically independent experiments; data are presented as Mean ± standard deviation.
**Figure S11.** (A) Analyses of single‐cell RNA sequencing data from the *Tabula Muris Consortium* show an enrichment of Spsb1 but not Spsb2, Spsb3, or Spsb4 in satellite cells and mesenchymal stem cells. (B) Provided legend for Cell Ontology Class showing satellite cells (pink) and mesenchymal stem cells (cyan).
**Figure S12.** C2C12 cells were transduced by control GFP, SPSB1 (WT) or mutants (SPSB‐Y129A, ‐TYAA or ‐ΔSOCS) retrovirus and differentiated for 5 days. (A) qRT‐PCR analysis of *Myog*, *Mymk*, *Mymx*, *Myh1*, *Myh3,* and *Myh7*. mRNA expression was normalized to *Gapdh*. (B) Western blot of lysates from above cells with anti‐Fast MyHC and anti‐Slow MyHC antibody. GAPDH was used as loading control. (C) Densitometric analysis of (B). Data were analysed with one‐way ANOVA followed by Tukey's post‐hoc test. * indicates significant differences between SPSB1 (wildtype or mutants as indicated) and the GFP control group, *p < 0.05, **p < 0.01, ***p < 0.001; # denotes a significant difference between indicated SPSB1 mutants and the SPSB1 wildtype group, #*p* < 0.05, ##*p* < 0.01, ###*p* < 0.001. *p < 0.05, **p < 0.01, ***p < 0.001. *n* = 3 biologically independent experiments; data are presented as Mean ± standard deviation.
**Figure S13.** (A) Western blot analysis of proteins isolated from undifferentiated C2C12 myoblasts (MB) and different stages of differentiation as indicated using anti‐TβRII and anti‐Fast MyHC antibody. GAPDH was used as loading control. (B) C2C12 cells were differentiated for 1, 3 and 5 days. qRT‐PCR analysis of *Tgfb1* at indicated timepoints. (C) Immunofluorescent staining of three‐ and five‐days differentiated C2C12 myotubes with anti‐TβRII (red) and anti‐Fast MyHC (green) antibodies. Nuclei were stained with DAPI (blue). (D) C2C12 cells were differentiated in the absence or presence of ITD‐1 (4 μM) for indicated timepoints. qRT‐PCR analysis of *Myog*, *Mymx*, and *Mymk* at indicated timepoints. (E) qRT‐PCR analysis of *Myh1*, *Myh3* and *Myh7* at indicated timepoints. mRNA expression was normalized to *Gapdh*. Data in (B) were analysed with one‐way ANOVA followed by Tukey's post‐hoc test; data in (D) and (E) were analysed with two‐way ANOVA followed by Tukey's post‐hoc test. * indicates a significant difference between ITD‐1‐ and vehicle‐treated cells, *p < 0.05, **p < 0.01, ***p < 0.001; *n* = 3 biologically independent experiments; data are presented as Mean ± standard deviation.
**Figure S14.** (A‐F) Cells were differentiated in the absence or presence of ITD‐1 (2 μM and 4 μM) for 5 days. Cell lysates were analysed by Western blot with anti‐ TβRII (A) and anti‐phospho Akt antibody (Ser473) (B), respectively. Total Akt was used as control for phospho Akt (Ser473) and GAPDH was used as loading control. Right panels show densitometric analysis of TβRII and phospho Akt (Ser473) abundance. (C) C2C12 myotubes described were incubated with OP‐puro labelling for 1 h. Red fluorescence (upper panel) corresponds to *de novo* synthesized polypeptides. Scale bar, 100 μm. (D) Immunofluorescent staining of C2C12 myotubes described with anti‐TβRII (red) and anti‐fast‐twitch MyHC (green) antibodies. Nuclei were stained with DAPI (blue). (E) Differentiation index, Fusion index, and Nuclei distribution in each myosin+ cell were quantified from images in panel (D). (F) Western blot analysis from cells lysates with antibodies as indicated. GAPDH was used as loading control. Data in (A), (B), (E; Differentiation and Fusion index), and (F) were analysed with one‐way ANOVA followed by Tukey's post‐hoc test; data in (E; myosin+ cells) were analysed with two‐way ANOVA followed by Tukey's post‐hoc test. * indicates a significant difference between ITD‐1‐ (2 μM and 4 μM) compared to vehicle‐treated cells, *p < 0.05, **p < 0.01, ***p < 0.001; # indicates a significant difference between both ITD‐1‐treated groups; i.e. 4 μM vs. 2 μM ITD‐1, #*p* < 0.05, ##*p* < 0.01, ###*p* < 0.001. *n* = 3 biologically independent experiments; data are presented as Mean ± standard deviation.
**Figure S15.** Cells were transduced by control GFP, SPSB1, Akt‐Myr, respectively, or co‐transduced by constitutive active Akt‐Myr and SPSB1 retrovirus and differentiated for 5 days. (A) qRT‐PCR analysis of *Myog*, *Mymk* and *Mymx* from cells differentiated for 1 day and *Myh1*, *3* and *7* (B) from cells differentiated for 5 days. mRNA expression was normalized to *Gapdh*. Data were analysed with one‐way ANOVA followed by Tukey's post‐hoc test. * indicates a significant difference between SPSB1‐, Akt‐Myr‐ or SPSB1 and Akt‐Myr‐treated groups compared with GFP control treated cells, *p < 0.05, **p < 0.01, ***p < 0.001; # indicates a significant difference between SPSB1‐ and SPSB1 + Akt‐Myr‐treated cells, #*p* < 0.05, ##*p* < 0.01, ###*p* < 0.001. *n* = 3 biologically independent experiments; data are presented as Mean ± standard deviation.
**Figure S16.** Primary myoblasts were transduced by control GFP, SPSB1, Akt‐Myr, respectively, or co‐transduced by Akt‐Myr and SPSB1 retrovirus and differentiated for 5 days. (A) Immunofluorescent staining of above cells with anti‐Fast MyHC as primary antibody and Alexa Fluor 555 conjugated secondary antibody (red). GFP (green) indicates retrovirally transduced cells. Scale bar, 100 μm. (B) Differentiation index, Fusion index, and Nuclei distribution in each myosin+ cell were quantified from images in panel (A). Data in (B; Differentiation and Fusion index) were analysed with one‐way ANOVA followed by Tukey's post‐hoc test; data in (B; myosin+ cells) were analysed with two‐way ANOVA followed by Tukey's post‐hoc test. * indicates a significant difference between SPSB1‐, Akt‐Myr‐ or SPSB1 and Akt‐Myr‐treated groups compared with GFP control treated cells, *p < 0.05, **p < 0.01, ***p < 0.001; # indicates a significant difference between SPSB1‐ and SPSB1 + Akt‐Myr‐treated cells, #*p* < 0.05, ##*p* < 0.01, ###*p* < 0.001. *n* = 3 biologically independent experiments; data are presented as Mean ± standard deviation.
**Figure S17.** (A) Primary myoblasts were transduced by control GFP, SPSB1, Akt‐Myr, respectively, or co‐transduced by Akt‐Myr and SPSB1 retrovirus and differentiated for 3 days. OP‐puro labelling was performed for 1 h. Red fluorescence corresponds to de novo synthesized polypeptides. (B) Primary myoblasts were transduced by control GFP, SPSB1, Myogenin, respectively, or co‐transduced by Myogenin and SPSB1 retrovirus and differentiated for 3 days. OP‐puro labelling was performed for 1 h. Red fluorescence corresponds to de novo synthesized polypeptides.
**Figure 18.** Eight‐week‐old male B6(C)/Rj‐Tyr^c/c^ mice were injected with 1*10^12^ vector genomes (vg) of AAV9 expressing shRNA_Spsb1 or control_shRNA. After 6 weeks mice were subjected to CLP or sham surgery. Analyses were performed 96 h after surgery (sham: control_shRNA, *n* = 6, shRNA_Spsb1: n = 6; CLP: control_shRNA, n = 6, shRNA_Spsb1: n = 6). (A) Experimental design. (B) qRT‐ PCR analysis of *Spsb1* expression in *tibialis anterior* and *gastrocnemius/plantaris* muscle. *p < 0.05, **p < 0.01; n.s. denotes not significant. Data are presented as Mean ± standard deviation. (C) Body weight and weights of *tibialis anterior* and *gastrocnemius/plantaris* muscle of CLP operated mice normalized to tibia length and expressed as relative change compared to sham operated mice. Data are presented as Mean ± standard deviation. *p < 0.05, ***p < 0.001. (D) Frequency distribution histograms of myofiber cross sectional area (MCSA) of Sham‐ and CLP‐treated control_shRNA and shRNA_Spsb1 mice of histological cross sections from *tibialis anterior* muscle. ***p < 0.001. (E, F) qRT‐PCR analysis of *Trim63* and *Fbxo32* expression in *tibialis anterior* (E) and *gastrocnemius/plantaris* muscle (F). *p < 0.05, **p < 0.01, ***p < 0.001. Data are presented as Mean ± standard deviation. n.s. denotes not significant.
**Figure S19.** Schematic model of SPSB1‐mediated inhibition of myogenesis by downregulating TGF‐β‐Akt‐Myogenin signalling. Under physiological conditions TGF‐β binds to TβRI and TβRII complex and activates the non‐canonical Akt and the canonical Smad pathway. TGF‐β promotes myogenesis via the non‐canonical TβRII‐Akt‐Myogenin pathway. Akt acts as a central regulator of transcription and translation, and increases protein synthesis, which is required for initiating myoblast fusion. Additionally, Akt induces expression of Myogenin/*Myog*, which in turn increases the expression of Myomaker/*Mymk*, Myomerger/*Mymx* as well as myosin heavy chain (MyHC) *Myh1, Myh3* and *Myh7*. An increase in proinflammatory cytokines, e.g., tumour necrosis factor (TNF), interleukin 1β (IL‐1β) and IL6, during sepsis cause an increase in Spsb1 expression by an activation of NF‐κB and gp130/JAK2/STAT3 signalling, respectively, in myocytes. SPSB1 targets TβRII and inhibits the TβRII‐Akt‐Myogenin pathway decreasing protein synthesis, myogenic fusion and differentiation contributing to impaired myogenic differentiation. Created with BioRender.com.
