# Supplementary material for: SPSB1‐mediated inhibition of TGF‐β receptor‐II impairs myogenesis in inflammation
Source: J Cachexia Sarcopenia Muscle. 2023 May 20;14(4):1721–36. doi: 10.1002/jcsm.13252 (PMC10401548; doi:10.1002/jcsm.13252)
Supplement: Supplementary file 1 — Table S1. Primers for generation of cDNA expression plasmids. Table S2. Primers for generation of retroviral expression plasmid. Table S3. Primers of site‐direct mutagenesis using SPSB1‐retroviral plasmid as template. Table S4. Primers for quantitative real‐time PCR. Figure S1. Quantitative RT‐PCR (qRT‐PCR) analysis of Spsb1, Spsb2, Spsb3, and Spsb4 from the tibialis anterior (TA), gastrocnemius plantaris (GP), soleus (Soleus), and extensor digitorum longus (EDL) muscle of 12‐week‐old male C57BL/6 J mice subjected to cecal ligation and puncture (CLP, n = 5, 24 h; n = 5–9, 96 h) or sham surgery (sham, n = 3, 24 h; n = 3–5, 96 h). Data in were analysed with two‐way ANOVA followed by Tukey's post‐hoc test. *p < 0.05, **p < 0.01, ***p < 0.001. Figure S2. Immunofluorescent staining of mouse TA muscle using anti‐SPSB1 (red), anti‐MyHC‐2A (green), anti‐MyHC‐2B (cyan), and anti‐Laminin (white) antibodies. Stars indicate enrichment of SPSB1 and in MyHC‐2A containing cells. Arrowheads reveal TβRII was absent on cytoplasmic membrane. Scale bar, 50 μm. Data in were analysed with two‐way ANOVA followed by Tukey's post‐hoc test. *p < 0.05, **p < 0.01, ***p < 0.001. Figure S3. qRT‐PCR of SPSB2 (A), SPSB3 (B), and SPSB4 (C) from the vastus lateralis muscle of patients with intensive care unit‐acquired weakness (ICUAW, n = 7) compared to healthy subjects (controls, n = 12). mRNA expression was normalized to GAPDH. Data were analysed with two‐tailed Student's t‐test. ***p < 0.001. Figure S4. (A) qRT‐PCR of Spsb1 from five‐day‐differentiated C2C12 myotubes (MT5) that were treated with TNF (10 ng/ml), IL‐1β (10 ng/ml) or IL6/IL6R (100 ng/ml) for indicated time points. (B) qRT‐PCR analysis of Spsb1 from five‐day‐differentiated C2C12 myotubes treated with SAA1 (10 ng/ml) or LPS (1 μg/mL) for 72 h. (C) qRT‐PCR analysis of Spsb1 from C2C12 myotubes that were treated with TGFβ (0.2 or 5 ng/ml) for 24 h and 72 h, as indicated. Figure S5. Immunofluorescent staining of TA muscle from sham or C [file JCSM-14-1721-s001.pdf]

SPSB1-mediated inhibition of TGF-β receptor-II impairs myogenesis in inflammation

Yi Li, Niklas Dörmann, Björn Brinschwitz, Melanie Kny, Elisa Martin, Kirsten Bartels, Ning Li, Priyanka Voori Giri, Stefan Schwanz, Michael Boschmann, Susanne Hille, Britta Fielitz, Tobias Wollersheim, Julius Grunow, Stephan B. Felix, Steffen Weber-Carstens, Friedrich C. Luft, Oliver J. Müller, Jens Fielitz

Online Supplementary Material

Online Supplementary Material

Animal Experiments

Animal model of polymicrobial sepsis

Cecal ligation and puncture (CLP) surgery was performed to induce polymicrobial sepsis as recently described [1-3]. Briefly, mice were anesthetized with isoflurane, placed on a heating plate to assure a constant body temperature of 37°C measured by a rectal probe. After shaving and disinfection of the abdominal skin, midline laparotomy was performed, the cecum was exposed and ligated using a non-absorbable surgical suture (Ethicon 6-0, Johnson & Johnson Medical GmbH, Umkirch, Germany). A 21-gauge needle was used to puncture the cecum once, and a small amount of cecum content was extruded. The cecum was then replaced into the abdominal cavity, and the incisions of the peritoneum and skin were closed with two separate layers of surgical sutures (Ethicon 6-0, Johnson & Johnson Medical GmbH, Umkirch, Germany). Sham mice were treated identically except for the ligation and puncture of the cecum. Directly after surgery 500 µl of sterile and prewarmed NaCl were applied by subcutaneous injection to all mice. Antibiotics were not administered in these experiments.

AAV9-mediated knockdown of *Spsb1* in vivo

An AAV vector genome plasmid was engineered for expression of a shRNA against Spsb1. For knock-down of Spsb1, we designed a shRNA duplex within a miR30 context as recently described[4]. The AAV genome plasmid pssMCKE-sh-

MmSpsb1-2-miR30hp-EGFP was generated by inserting the Spsb1 shRNA (mature antisense: 5'-TTGGTCTTTCTAATCCTGCCTT-3') [5] 3′ of an EGFP reporter gene under control of the muscle creatine kinase promoter (MCKE). Accordingly, pssMCKE-sh-ctrl-miR30hp-EGFP was designed as a non-silencing control (mature antisense: 5'-CTTACTCTCGCCCAAGCGAGAG-3'). For AAV9 production, each genome plasmid together with the adenoviral helper plasmid pDP9rs, coding for the AAV serotype 9 capsid and all relevant adenoviral helper factors, was co-transfected into low passage HEK293T cells and processed as described previously [5]. $1*10^{12}$ vector genomes (vg) were injected into the tail vein of 8-week-old male B6(C)/Rj-$Tyr^{c/c}$ mice. Six weeks after AAV9 injection CLP or sham surgery was performed. Ninety-six hours after surgery the experiment was terminated and muscle tissues were harvested.

Histological analyses and measurement of myocyte cross-sectional area.

Tibialis anterior and *gastrocnemius/plantaris* were obtained from mice following sham or CLP surgery. Muscles were flash frozen in liquid nitrogen with gum tragacanth (Merck, Germany) as cryoprotectant. A Leica cryotome CM3050S (Leica Microsystems GmbH, Germany) was used to cut histological cross sections of TA with a thickness of 5 μm. Sections were stained with Hematoxylin & Eosin (H&E) as described earlier [6, 7]. To analyze the myocyte cross sectional area (MCSA) images were acquired with Leica CTR 6500 HS microscope and the Leica digital camera DFC 425 (Leica Microsystems GmbH, Germany). Image J software 1.51v9 software (Wayne

Rasband, National Institutes of Health, USA) was used to measure 100 MSCAs per mouse and condition [3, 8-11]. MCSA measurements were performed in control_shRNA (sham n=6, CLP n=6) and Spsb1_shRNA treated mice (sham n=6, CLP n=6). The person who performed measurements was blinded to the specific treatment.

Immunohistochemical analysis of skeletal muscle.

Immunohistochemistry was performed on 8 μm thin cryo-sections as recently published[1, 2]. Primary antibodies: SC-71 (MyHC-2A, IgG1, 1:100;), BF-F3 (MyHC-2B, IgM, 1:100,), laminin beta-1 (1:100; Abcam), SPSB1 (1:100, Life Technologies, USA), TβRII (1:100, Cell Signaling, UK). Secondary antibodies: donkey-anti-mouse-Alexa Fluor 405, goat-anti-mouse-Alexa Fluor 488, goat-anti-mouse-Alexa Fluor 549, goat-anti-rat-Alexa Fluor 647 (all 1:100, Life Technologies, USA).

Cell culture

C2C12 myoblasts (ATCC, USA) were cultivated in growth medium (GM: Dulbecco's Modified Eagle's medium (DMEM; 4.5g/L glucose, Fisher Scientific, USA), 10% fetal bovine serum (FBS, Biochrom GmbH, Germany), 2mM glutamine, 1U/ml penicillin, 1μg/ml streptomycin (all Sigma Aldrich, Germany)) at 37°C in a 5% CO_2 atmosphere. For differentiation, myoblasts were transferred to differentiation medium (DM: DMEM, 1g/L glucose, Sigma Aldrich, Germany, 2% FBS). C2C12 cells were transfected with 1μg of expression plasmids per well in a six-well plate using Lipofectamine™ 3000 Reagent (Life Technologies, USA) according to the manufacturer’s protocol. For siRNA transfection, 50nM siRNA (Dharmacon; control

siRNA D-001810-10-05, Il6st siRNA, J-040007-09-0005) was transfected using Dharmafect3 (Dharmacon) according to the manufacturer's protocol.

Isolation of primary myoblasts: Isolation of primary skeletal muscle myoblasts was performed as recently published[12]. Briefly, 6-8 weeks old mice were sacrificed by cervical dislocation and hindlimb muscles were dissected and collected in sterile ice-cold 1x PBS. Muscle tissue was minced and digested by adding digestion buffer (DMEM containing 1.0g/L glucose, collagenase II 1,15mg/ml, dispase II 1,6mg/ml) for 1h at 37°C in a water bath. Following centrifugation at 1500g for 10 min the cell pellet was dissolved in plating medium (DMEM containing 1.0g/L glucose, 10% horse serum) and filtered through a 40µm Cell Strainer, following another round of centrifugation the cell pellet was again dissolved in plating medium and the cell suspension was preplated on a 10 cm dish for 2 h at 37°C in a CO_2 Incubator. The cell containing medium was then removed from the plate and centrifuged at 1500g for 3 min and the resulting pellet was then dissolved in 1 ml growth medium (400ml DMEM, 1.0 g/L glucose, 20% FBS, 10% horse serum, 2.5ng/ml bFGF, 1% Penicillin/Steptomycin). 50,000 cells were plated onto a Geltrex coated 8-well IBIDI slide. Two days after isolation the growth medium was changed and three days after isolation growth medium was changed to differentiation medium (DMEM, 1.0g/L glucose, 2% horse serum, 1x Penicillin/Steptomycin).

Commercial Mouse skeletal muscle myoblasts: Mouse skeletal muscle myoblasts originated from mouse GP and TA muscle were purchased from iXCells Biotechnologies USA, Inc. (Catalog Number: 10MU-033). Cells were cultivated on

Geltrex coated plates or IBIDI slides in growth medium (Catalog Number: MD-0064) and once they reached 70-80% confluency growth medium was replaced by differentiation medium (DMEM, 1.0g/L Glucose, 2% Horse Serum, 1x Penicillin/Steptomycin) to initiate differentiation.

Immunofluorescent imaging, and analysis of myogenic differentiation

Myoblasts were seeded onto coverslips or μ-Slide 8-Well coverslips (IBIDI cell in focus, Germany). After treatment cells were fixed with 4% paraformaldehyde, permeabilized by 0.1% Triton X-100 and blocked with 5% goat serum. Primary antibodies: anti-FLAG (F3165, 1:500, Sigma Aldrich, Germany), anti-Myc (06-549, 1:500, Millipore, USA), anti-Smad3 (#9520, 1:100, Cell Signaling, UK), anti-TβRII (ab186838, 1:500, Abcam, UK), anti-Fast Myosin (clone My32, M4276, 1:500, Millipore, USA). Secondary antibodies: goat-anti-mouse-Alexa Fluor 488, goat-anti-mouse-Alexa Fluor 555, goat-anti-rabbit-Alexa Fluor 488, goat-anti-rabbit-Alexa Fluor 555 (1:500, Life Technologies, USA). 4′,6-Diamidin-2-phenylindol (DAPI) was used for nuclear staining. Samples were mounted with ProLongGold Antifade Reagent (Life Technologies, USA). The Leica CTR 6500 fluorescence microscope, the Leica DFC 360 FX digital camera (Leica, Germany) and the Zeiss LSM 700 confocal microscope (Carl Zeiss, Germany) were used for imaging, respectively. Images were analyzed with Zen 2009 (Carl Zeiss, Germany) or FIJI/ImageJ software (NIH, USA).

Analysis of myogenic differentiation: the differentiation index was calculated as the

percentage of nuclei in myosin positive (myosin$^+$) cells related to the total number of nuclei per field of view. Fusion index was quantified by the percentage of nuclei contained in myotubes (a myosin$^+$ cell with at least two nuclei) related to the total number of nuclei per field of view. For each condition shown, 30 fields of view (>360 myosin$^+$ cells) were quantified.

Cytokine and inhibitor treatment

C2C12 cells were differentiated into myotubes for 5 days and then treated with TNF (10 ng/ml, eBioscience, USA), IL6/IL6R (100 ng/ml, R&D Systems, USA), IL-1β (25 ng/ml, Prospec, Israel), recombinant human Apo-serum amyloid A1 (SAA1; 10 μg/ml, Peprotech, USA) or lipopolysaccharide (LPS; 1 μg/mL, from Escherichia coli O111:B4, Merck, Germany) for indicated time points. The vehicle 0.1% BSA in PBS was used as a control. For pathway analyses, five days differentiated C2C12 myotubes were treated with a JAK2 inhibitor (AG490; 10μM, Sigma-Aldrich, MO, USA), a STAT3 inhibitor (C188-9; 10μM, Merck-Milipore, Germany) or an IKK-inhibitor BMS-345541 (5 μM, Abcam, UK) for 60 min prior to 2 h of IL-6 and TNF or IL-1β treatment, as indicated. To detect phosphorylation of Akt, cells were transduced with SPSB1 or GFP control expressing-retrovirus and differentiated for 5 days. Cells were then treated with recombinant human TGF-β1 (5 ng/ml, PeproTech, USA) or the same volume of vehicle (10mM citric acid dissolved in differentiation medium) for 5 min.

For analysis of differentiation in response to ITD-1, DMEM was supplemented with ITD-1 (2 μM or 4 μM, Ethyl 4-([1,1'-biphenyl]-4-yl)-2,7,7-trimethyl-5-oxo-

1,4,5,6,7,8-hexahydroquinoline-3-carboxylate, Tocris Bioscience, UK) or DMSO as vehicle. Differentiation medium including inhibitor or vehicle was exchanged every 24 h during differentiation and differentiation was monitored for 5 days. On differentiation day 1, 3 and 5, cells were harvested for RNA or protein analysis. For morphological analyses, cells were seeded onto coverslips, differentiated, and treated as indicated, and analyzed with immunofluorescent staining.

cDNA expression plasmids, retrovirus production and transduction of cells

The coding sequences (CDS) of *Spsb1*, *Tgfbr2* and *Myog* were PCR amplified from mouse TA cDNA (primers listed in Table S1) and cloned into pcDNA™3.1-FLAG or pcDNA™3.1-Myc (both Invitrogen™, Life Technologies, USA). FLAG-SPSB1, Myogenin-Myc and myristylated Akt (pBSFI-Akt-Myr; Addgene)[13] expression plasmids were used to generate retroviral expression plasmids (pMP71-IRES-GFP-FLAG-SPSB1, pMP71-IRES-GFP-Myogenin-Myc, pMP71-IRES-GFP-HA-Akt-Myr) (primers listed in Table S2). SPSB1 mutants (Y129A; T160A/Y161A (TYAA), ΔSOCS) were generated using Q5® Site-Directed Mutagenesis Kit (New England Biolab, USA) (primers listed in Table S3). All constructs were sequence verified. Platinum-E (Cell Biolabs, USA) cells were transfected with retroviral constructs using Lipofectamine™ 3000 according to the manufacturer's protocol. Retroviruses were harvested 48h after transfection and myocytes were transduced by spinoculation.

RNA isolation and real-time PCR analysis

Total RNA was isolated from cultured cells using TRIzol® reagent (Invitrogen, Life Technologies Corporation, USA) and cDNA was synthesized using the SuperScript® First-Strand Synthesis kit (Invitrogen) following the manufacturer's protocol and as published previously[1, 14]. RNA expression was analyzed by quantitative real-time polymerase chain reaction (qRT-PCR) using primers (see Table S4) and SYBR Green PCR Master Mix (Roche, Switzerland). qRT-PCR reactions were performed in a Step-One™ Plus thermocycler (Applied Biosystems, USA) using a cDNA standard curve. Gene expression was normalized to the expression levels of the stably expressed reference gene *glyceraldehyde-3-phosphate dehydrogenase* (*Gapdh*).

Protein extraction and Western blot analysis

Control cells or cells transfected with expression plasmids were lysed in ice-cold extraction buffer (150 mM NaCl, 50 mM Tris-HCl, 1 mM EDTA (sodium salt), 0.5% (w/v) sodium deoxycholate, 1% (v/v) NonidetP40, 0.1% (w/v) SDS, PhosSTOP and protease inhibitors (CompleteTM, Roche, Switzerland), pH 8.0). Lysates were denatured using SDS sample buffer (50 mM Tris-HCl, 2% SDS, 6% glycerol, 5 % β-mercaptoethanol, 0.1% bromophenol blue). Proteins were separated by 10-15% SDS-PAGE and transferred onto Amersham Hybond P 0.45µm PVDF membranes (VWR, USA). Membranes were blocked with 5% BSA/TBST or 5% Milk/TBST dependent on the antibodies used and were then incubated with the indicated primary antibodies at 4°C overnight, followed by incubation with anti-horseradish peroxidase (HRP) conjugated secondary antibodies (Cell Signaling, UK). Primary antibodies: anti-

DYKDDDDK FLAG (#2368), anti-Akt (#9272), anti-pAkt (Ser473, #4060), anti-TβRII (#79424), anti-HA-Tag (3724) (all 1:1000, from Cell signaling, UK); anti-Fast MyHC (clone My32, M4276), anti-slow MyHC (clone NOQ7, M8421), anti-Myogenin (M5815) (all 1:1000, from Sigma Aldrich, Germany); anti-Myc (06-549, 1:500), anti-glyceraldehyde-3-phosphate dehydrogenase (GAPDH; MAP374, 1:5000) (both from Millipore, USA); anti-HA (M180-3, 1:1000, MBL Life Science, Japan); and anti-SPSB1 (PA5-89504, 1:1000, Thermo Fisher Scientific, USA). Detection was performed using SuperSignal™ West Pico Chemiluminescent Substrate (Thermo Fisher Scientific, USA) according to the manufacturer's instructions. The stably expressed GAPDH was used as loading control for all the proteins. Total Akt was used as control for pAkt (Ser473).

Analysis of protein synthesis.

Protein synthesis was determined with the Global Protein Synthesis Assay Kit (ab235634, Abcam, UK) according to the manufacturers protocol. Briefly, $1.2x10^4$ C2C12 cells and $1.8x10^4$ primary myoblasts were plated onto μ-Slide 8-Well coverslips (IBIDI cell in focus, Germany), respectively. After 24h C2C12 cells were transduced with retroviruses and differentiated for 5 days as described. Primary myoblasts were transduced after 72h and differentiated for 3 days. On differentiation day 3 (primary myoblasts) and day 5 (C2C12 cells), as indicated, cells were washed with PBS and incubated with fresh differentiation medium containing cell-permeable O-Propargyl-puromycin (OP-puro) protein label for 1h at 37°C. As a negative control, cells were pre-treated with cycloheximide (CHX, 50 μg/ml) or PBS as control for 30

min prior to the analyses. Reaction was stopped by removing the medium. Cells were then fixed, permeabilized and incubated with the provided reagents for 30 min at room temperature. The alkyne contained in the OP-puro protein label reacted with the fluorescent azide in the reaction cocktail. 4′,6-Diamidin-2-phenylindol (DAPI) was used to label the cell nuclei. Experiments were performed at least 3 times.

Co-Immunoprecipitation (Co-IP)

C2C12 cells were transfected with expression plasmids or transduced with retrovirus, as indicated. After 48h, cells were harvested in lysis buffer (150mM Phosphate, 150mM NaCl, protease inhibitor, pH7.4) and 10% of the lysates were used for input controls. Lysates were immunoprecipitated (IP) with anti-FLAG M2 affinity gel (A2220, Sigma Aldrich, Germany). Lysates and immunoprecipitates were subjected to Western blot analyses with the indicated antibodies.

Cycloheximide chase assay

Cycloheximide (CHX) chase assay was performed in 1 day differentiated C2C12 cells 1, 3 and 6h after CHX treatment. Protein contents were analyzed by immunoblotting.

Ubiquitination assay

COS-7 cells were transfected as indicated. After 42h cells were treated with MG132 (25μM) or DMSO (0.25%, vehicle) for a further 6h and then lysed in lysis buffer (50mM Tris, 150mM NaCl, 1% Triton X-100, 50mM NaF, 2mM $MgCl_2$, 1mM Na_3VO_4, 25μg/ml leupeptin, 25mM N-Ethylmaleimide (Sigma Aldrich, Germany), 25μg/ml aprotinin) and 10% of the lysates were used for input controls. Lysates were

immunoprecipitated (IP) with anti-FLAG M2 affinity gel (A2220, Sigma Aldrich, Germany). Lysates and immunoprecipitates were subjected to Western blot analyses with the indicated antibodies.

Supplementary Tables

Table S1. Primers for generation of cDNA expression plasmids.

Primer Name (restriction site)	Oligonucleotide sequence (5' → 3') (restriction site underlined)
FLAG-SPSB1-F (EcoRI)	CTCATCGAATTCATGGGTCAGAAGGTCACAGG
FLAG-SPSB1-R (NotI)	TCATCGCGGCCGCTCACTGGTAGAGGAGGTAGGCT
SPSB1-Myc-F (NotI)	ATCGCGGCCGCAATGGGTCAGAAGGTCACAGG
SPSB1-Myc-R (EcoRI)	ATCGAATTCGCTGGTAGAGGAGGTAGGCTTTG
FLAG-TβRII(/ΔExon2)-F (EcoRI)	CATCCGAATTCATGGGTCGGGGGCTGCTC
FLAG-TβRII(/ΔExon2)-R (NotI)	CATCGCGGCCGCCTATTTGGTAGTGTTCAGCGAGC

F: forward; R: reverse; SPSB1: splA/ryanodine receptor (SPRY) domain and SOCS-box containing protein 1; TβRII: transforming growth factor β type II receptor.

Table S2. Primers for generation of retroviral expression plasmid.

Primer Name (restriction site)	Oligonucleotide sequence (5' → 3') (restriction site underlined)
FLAG-SPSB1-F (NotI)	GCGCGCGGCCGCATGGACTACAAAGACG
FLAG-SPSB1-R (NotI)	TCATCGCGGCCGCTCACTGGTAGAGGAGGTAGGCT
Myogenin-F (NotI)	GAATGCGGCCGCATGGAGCTGTATGAGACATCCC
Myogenin+Myc-overlap-R	TGAGATGAGTTTTTGTTCGTTGGGCATGGTTTCGT
Myogenin+Myc-overlap-F	GACGAAACCATGCCCAACGAACAAAAACTCATCTC
Myc-R (EcoRI)	CCGGAATTCCTCAATGATGATGATGATGATGGTCGA

F: forward; R: reverse.

Table S3. Primers of site-direct mutagenesis using SPSB1-retroviral plasmid as template.

**Primer Name**	**Oligonucleotide sequence (5' → 3')**
FLAG-SPSB1-Y129A-F	CAAAGTTGGGGCCACACGTGGACTGCATG
FLAG-SPSB1-Y129A-R	CCCCTGATGGCGTCCGTG
FLAG-SPSB1-TYAA-F	GCCAAGTAAAGCCGCCCCAGCCTTTCTGGAG
FLAG-SPSB1-TYAA-R	TGGTTCTTGCCGTCGTGG
FLAG-SPSB1-ΔSOCS-F	TGAGCGGCCGCTAAGCTT
FLAG-SPSB1-ΔSOCS-R	ATCAAGTCCGTTCAAGTAGCGC

F: forward; R: reverse.

239 **Table S4. Primers for quantitative real-time PCR.**

Primer Name	Oligonucleotide sequence (5' → 3')
Mm_Spsb1-F	GGTCAGAAGGTCACAGGAGG
Mm_Spsb1-R	GTGATCTGCCATACATGCAGTC
Mm_Spsb2-F	AAGAAGAGTGGAGGAACCACAAT
Mm_Spsb2-R	CAAAGGCAGAGTGGATATTTGAC
Mm_Spsb3-F	GCAGCTCTAACTGGGGCTATGACTC
Mm_Spsb3-R	ACAGGCACAGCACTGGGGATGGATG
Mm_Spsb4-F	GAGTGCTGTGTGGGGTCA
Mm_Spsb4-R	AGGGCTGAGCGGATGGAT
Mm_Il6st-F	CCCATGGGCAGGAATATAGA
Mm_Il6st-R	CATAATCCAAGATTTTCCCATTG
Mm_Fbxo32-F	AGTGAGGACCGGCTACTGTG
Mm_Fbxo32-R	GATCAAACGCTTGCGAATCT
Mm_Gapdh-F	ATGGTGAAGGTCGGTGTGA
Mm_Gapdh-R	AATCTCCACTTTGCCACTGC
Mm_Myog-F	GCGATCTCCGCTACAGAGG
Mm_Myog-R	GCTGTGGGAGTTGCATTCA
Mm_Myod-F	AGCACTACAGTGGCGACTCA
Mm_Myod-R	GGCCGCTGTAATCCATCA
Mm_Myomaxin-F	CCGTCGGATGTCAAGACAAC
Mm_Myomaxin-R	GAGAGTAGAGGTCTTCCAAGG
Mm_Mymk-F	ATCGCTACCAAGAGGCGTT
Mm_Mymk-R	CACAGCACAGACAAACCAGG
Mm_Mymx-F	CAGGAGGGCAAGAAGTTCAG
Mm_Mymx-R	ATGTCTTGGGAGCTCAGTCG
Mm_Myh1-F	AATCAAAGGTCAAGGCCTACAA
Mm_Myh1-R	GAATTTGGCCAGGTTGACAT
Mm_Myh3-F	GGATGGGAAAGTCACTGTGG
Mm_Myh3-R	GTCCTCTGGCTTAACCACCA
Mm_Myh4-F	TGGCCGAGCAAGAGCTAC
Mm_Myh4-R	TTGATGAGGCTGGTGTTCTG
Mm_Myh7-F	CGCATCAAGGAGCTCACC
Mm_Myh7-R	CTGCAGCCGCAGTAGGTT
Mm_Smad7-F	TGCAAAGTGTTCAGGTGGCCG
Mm_Smad7-R	ATCCCCAGGCTCCAGAAGAAG
Mm_Tgfb1-F	TGGAGCAACATGTGGAACTC
Mm_Tgfb1-R	GTCAGCAGCCGGTTACCA
Mm_Trim63-F	CCTGCAGAGTGACCAAGGA
Mm_Trim63-F	GGCGTAGAGGGTGTCAAACT
Hs_SPSB1-F	AGTACATGGGAGTGGCTTTTC
Hs_SPSB1-R	ACAAATCCATGAGCGGCAG
Hs_SPSB2-F	ACCCTCTATCCGGCAGTAAG

Hs_SPSB2-R	GGTGCAGAAGGGAGTGTG
Hs_SPSB3-F	TCTAGCAGGCTCCACTAACT
Hs_SPSB3-R	CCCAGCACAGTCACAGAAG
Hs_SPSB4-F	CGAGGTCTCAAGGGCAAG
Hs_SPSB4-R	GGCACAGGTCCATCAGTG
Hs_GAPDH-F	GAAGGTGAAGGTCGGAGTCA
Hs_GAPDH-R	AATGAAGGGGTCATTGATGG

Mm indicates Mus musculus; Hs, Homo sapiens; F, forward; R, reverse; Fbxo32 indicates F-Box Protein 32 (Atrogin1); Gapdh/GAPDH, Glyceraldehyde-3-phosphate dehydrogenase; Il6st, interleukin 6 signal transducer (also known as glycoprotein 130 (gp130)); Myog, Myogenin; Mymk, Myomaker; Mymx, Myomerger; Myh, Myosin heavy chain; Spsb1/SPSB1, splA/ryanodine receptor (SPRY) domain and SOCS-box containing protein 1; Smad7, homologues of the Drosophila protein mothers against decapentaplegic and the Caenorhabditis elegans protein 7; Tgfb1, transforming growth factor β1; Trim63, Tripartite Motif Containing 63 (MuRF1).

Supplementary Figures

Figure S1. Quantitative RT-PCR (qRT-PCR) analysis of *Spsb1*, *Spsb2*, *Spsb3*, and *Spsb4* from the tibialis anterior (TA), gastrocnemius plantaris (GP), soleus (Soleus), and extensor digitorum longus (EDL) muscle of 12-week-old male C57BL/6J mice subjected to cecal ligation and puncture (CLP, n = 5, 24 h; n = 5-9, 96 h) or sham surgery (sham, n = 3, 24 h; n = 3-5, 96 h). Data in were analyzed with two-way ANOVA followed by Tukey's post-hoc test. *$p < 0.05$, **$p < 0.01$, ***$p < 0.001$.

Figure S2. Immunofluorescent staining of mouse TA muscle using anti-SPSB1 (red), anti-MyHC-2A (green), anti-MyHC-2B (cyan), and anti-Laminin (white) antibodies. Stars indicate enrichment of SPSB1 and in MyHC-2A containing cells. Arrowheads reveal TβRII was absent on cytoplasmic membrane. Scale bar, 50 μm. Data in were analyzed with two-way ANOVA followed by Tukey's post-hoc test. *$p < 0.05$, **$p < 0.01$, ***$p < 0.001$.

Figure S3. qRT-PCR of *SPSB2* (A), *SPSB3* (B), and *SPSB4* (C) from the *vastus lateralis* muscle of patients with intensive care unit-acquired weakness (ICUAW, n = 7) compared to healthy subjects (controls, n = 12). mRNA expression was normalized to *GAPDH*. Data were analyzed with two-tailed Student's *t*-test. ***$p < 0.001$.

Figure S4. (A) qRT-PCR of *Spsb1* from five-day-differentiated C2C12 myotubes (MT5) that were treated with TNF (10 ng/ml), IL-1β (10 ng/ml) or IL6/IL6R (100 ng/ml) for indicated time points. (B) qRT-PCR analysis of *Spsb1* from five-day-differentiated

C2C12 myotubes treated with SAA1 (10 ng/ml) or LPS (1 μg/mL) for 72 h. (C) qRT-PCR analysis of *Spsb1* from C2C12 myotubes that were treated with TGFβ (0.2 or 5 ng/ml) for 24 h and 72 h, as indicated.

Figure S5. Immunofluorescent staining of TA muscle from sham or CLP operated mice after 96 h using anti-TβRII (red) and anti-MyHC-2A (green) antibody. Nuclei were stained with DAPI (blue). Arrowheads indicate reduction of TβRII at the cytoplasmic membrane. Scale bar, 100 μm.

Figure S6. Heat map of significantly regulated genes ($p < 0.05$) contained in Gene Ontology (GO) term analysis (Biological process) "cellular response to transforming growth factor beta stimulus" (GO:0071560), where they were significantly enriched (p = 8.84E-05, FDR 0.002), in tibialis anterior muscle of septic wildtype mice 24 h and 96 h after surgery (n = 3 for each condition).

Figure S7. (A) Heat map of significantly regulated genes ($p < 0.05$) contained in Kyoto Encyclopedia of Genes and Genomes (KEGG)-pathway "Transforming growth factor-beta signaling pathway" (mmu04350), where they were significantly enriched (p = 5.4E-04, FDR 0.0038), in tibialis anterior muscle of septic wildtype mice 24 h and 96 h after surgery (n = 3 for each condition). (B) Position of significantly regulated genes in KEGG-pathway mmu04350. Regulated genes are shown in red.

Figure S8. (A) Subcellular distribution of SPSB1-Myc, FLAG-TβRII or FLAG-TβRII-ΔEx2 separately transfected C2C12 cells as detected by immunofluorescence using

anti-Myc antibody together with A555-coupled secondary antibody (red) or anti-FLAG antibody together with A488-coupled secondary antibody (green). (B) Subcellular distribution and co-localization of endogenous TβRII and SPSB1-Myc in transfected C2C12 cells. Anti-TβRII together with A488-coupled secondary antibody (green) and anti-Myc antibody together with A555-coupled secondary antibody (red) were used. Nuclei were stained with DAPI (blue). Scale bar, 20 μm. (C) Cells were transduced with a retrovirus encoding GFP (control), SPSB1 or SPSB1-ΔSOCS for 48 h and then treated with cycloheximide (CHX, 50 μg/ml) as indicated. Western blot analysis of cell lysates. Anti-FLAG antibody shows over-expressed FLAG-SPSB1. GAPDH was used as loading control. Densitometric analysis.

Figure S9. C2C12 cells were transduced by control GFP, SPSB1 (WT) or mutants (SPSB1-Y129A, -TYAA or -ΔSOCS) retrovirus and differentiated for 5 days**.** (A) Immunofluorescence of Smad3 (red) and DAPI (blue). Arrowheads shows co-localization of Smad3 and nuclei in GFP and SPSB1-mutant transduced cells. Scale bar, 20 μm. (B) Quantification of the percentage of Smad3 positive ($Smad3^{+}$) nuclei. (C) qRT-PCR analysis of TGF-β-Smad3 responsive gene *Smad7*. Data were analyzed with one-way ANOVA followed by Tukey's post-hoc test. * indicates significant differences between SPSB1 (wildtype or mutants as indicated) and the GFP control group, $*p < 0.05$, $**p < 0.01$, $***p < 0.001$; $^{\#}$ denotes a significant difference between indicated SPSB1 mutants and the SPSB1 wildtype group, $^{\#}p < 0.05$, $^{\#\#}p < 0.01$, $^{\#\#\#}p < 0.001$; n = 3 biologically independent experiments; data are presented as Mean ± standard deviation.

Figure S10. C2C12 cells were transduced with control GFP or SPSB1 retrovirus and differentiated for 1, 3 or 5 days. (A) qRT-PCR analysis of *Spsb1* from C2C12 cells at indicated timepoints. (B) Direct imaging of transduced C2C12 cells at indicated timepoints. GFP signals denote transduced cells. Scale bar, 100 μm. (C) qRT-PCR analysis of *Myog*, *Mymk*, *Mymx*, *Myh1*, *Myh3,* and *Myh7*. mRNA expression was normalized to *Gapdh*. Data were analyzed with two-way ANOVA followed by Tukey's post-hoc test. *$p < 0.05$, **$p < 0.01$, ***$p < 0.001$. n = 3 biologically independent experiments; data are presented as Mean ± standard deviation.

Figure S11. (A) Analyses of single-cell RNA sequencing data from the *Tabula Muris Consortium* show an enrichment of Spsb1 but not Spsb2, Spsb3, or Spsb4 in satellite cells and mesenchymal stem cells. (B) Provided legend for Cell Ontology Class showing satellite cells (pink) and mesenchymal stem cells (cyan).

Figure S12. C2C12 cells were transduced by control GFP, SPSB1 (WT) or mutants (SPSB-Y129A, -TYAA or -ΔSOCS) retrovirus and differentiated for 5 days. (A) qRT-PCR analysis of *Myog*, *Mymk*, *Mymx*, *Myh1*, *Myh3,* and *Myh7*. mRNA expression was normalized to *Gapdh*. (B) Western blot of lysates from above cells with anti-Fast MyHC and anti-Slow MyHC antibody. GAPDH was used as loading control. (C) Densitometric analysis of (B). Data were analyzed with one-way ANOVA followed by Tukey's post-hoc test. * indicates significant differences between SPSB1 (wildtype or mutants as indicated) and the GFP control group, *$p < 0.05$, **$p < 0.01$, ***$p < 0.001$; $^{\#}$ denotes a significant difference between indicated SPSB1 mutants and the SPSB1

wildtype group, $^{\#}p < 0.05$, $^{\#\#}p < 0.01$, $^{\#\#\#}p < 0.001$. $^{*}p < 0.05$, $^{**}p < 0.01$, $^{***}p < 0.001$. n = 3 biologically independent experiments; data are presented as Mean ± standard deviation.

Figure S13. (A) Western blot analysis of proteins isolated from undifferentiated C2C12 myoblasts (MB) and different stages of differentiation as indicated using anti-TβRII and anti-Fast MyHC antibody. GAPDH was used as loading control. (B) C2C12 cells were differentiated for 1, 3 and 5 days. qRT-PCR analysis of *Tgfb1* at indicated timepoints. (C) Immunofluorescent staining of three- and five-days differentiated C2C12 myotubes with anti-TβRII (red) and anti-Fast MyHC (green) antibodies. Nuclei were stained with DAPI (blue). (D) C2C12 cells were differentiated in the absence or presence of ITD-1 (4μM) for indicated timepoints. qRT-PCR analysis of *Myog*, *Mymx*, and *Mymk* at indicated timepoints. (E) qRT-PCR analysis of *Myh1*, *Myh3* and *Myh7* at indicated timepoints. mRNA expression was normalized to *Gapdh*. Data in (B) were analyzed with one-way ANOVA followed by Tukey's post-hoc test; data in (D) and (E) were analyzed with two-way ANOVA followed by Tukey's post-hoc test. * indicates a significant difference between ITD-1- and vehicle-treated cells, $^{*}p < 0.05$, $^{**}p < 0.01$, $^{***}p < 0.001$; n = 3 biologically independent experiments; data are presented as Mean ± standard deviation.

Figure S14. (A-F) Cells were differentiated in the absence or presence of ITD-1 (2μM and 4μM) for 5 days. Cell lysates were analyzed by Western blot with anti-TβRII (A) and anti-phospho Akt antibody (Ser473) (B), respectively. Total Akt was

used as control for phospho Akt (Ser473) and GAPDH was used as loading control. Right panels show densitometric analysis of TβRII and phospho Akt (Ser473) abundance. (C) C2C12 myotubes described were incubated with OP-puro labelling for 1 h. Red fluorescence (upper panel) corresponds to *de novo* synthesized polypeptides. Scale bar, 100 µm. (D) Immunofluorescent staining of C2C12 myotubes described with anti-TβRII (red) and anti-fast-twitch MyHC (green) antibodies. Nuclei were stained with DAPI (blue). (E) Differentiation index, Fusion index, and Nuclei distribution in each myosin$^+$ cell were quantified from images in panel (D). (F) Western blot analysis from cells lysates with antibodies as indicated. GAPDH was used as loading control. Data in (A), (B), (E; Differentiation and Fusion index), and (F) were analyzed with one-way ANOVA followed by Tukey`s post-hoc test; data in (E; myosin$^+$ cells) were analyzed with two-way ANOVA followed by Tukey's post-hoc test. * indicates a significant difference between ITD-1- (2 µM and 4 µM) compared to vehicle-treated cells, *$p < 0.05$, **$p < 0.01$, ***$p < 0.001$; $^{\#}$ indicates a significant difference between both ITD-1-treated groups; i.e. 4 µM vs. 2 µM ITD-1, $^{\#}p < 0.05$, $^{\#\#}p < 0.01$, $^{\#\#\#}p < 0.001$. n = 3 biologically independent experiments; data are presented as Mean ± standard deviation.

Figure S15. Cells were transduced by control GFP, SPSB1, Akt-Myr, respectively, or co-transduced by constitutive active Akt-Myr and SPSB1 retrovirus and differentiated for 5 days. (A) qRT-PCR analysis of *Myog*, *Mymk* and *Mymx* from cells differentiated for 1 day and *Myh1*, *3* and *7* (B) from cells differentiated for 5 days. mRNA expression was normalized to *Gapdh*. Data were analyzed with one-way ANOVA followed by

Tukey's post-hoc test. * indicates a significant difference between SPSB1-, Akt-Myr- or SPSB1 and Akt-Myr-treated groups compared with GFP control treated cells, *$p < 0.05$, **$p < 0.01$, ***$p < 0.001$; # indicates a significant difference between SPSB1- and SPSB1 + Akt-Myr-treated cells, #$p < 0.05$, ##$p < 0.01$, ###$p < 0.001$. n = 3 biologically independent experiments; data are presented as Mean ± standard deviation.

Figure S16. Primary myoblasts were transduced by control GFP, SPSB1, Akt-Myr, respectively, or co-transduced by Akt-Myr and SPSB1 retrovirus and differentiated for 5 days. (A) Immunofluorescent staining of above cells with anti-Fast MyHC as primary antibody and Alexa Fluor 555 conjugated secondary antibody (red). GFP (green) indicates retrovirally transduced cells. Scale bar, 100 μm. (B) Differentiation index, Fusion index, and Nuclei distribution in each myosin$^+$ cell were quantified from images in panel (A). Data in (B; Differentiation and Fusion index) were analyzed with one-way ANOVA followed by Tukey`s post-hoc test; data in (B; myosin$^+$ cells) were analyzed with two-way ANOVA followed by Tukey's post-hoc test. * indicates a significant difference between SPSB1-, Akt-Myr- or SPSB1 and Akt-Myr-treated groups compared with GFP control treated cells, *$p < 0.05$, **$p < 0.01$, ***$p < 0.001$; # indicates a significant difference between SPSB1- and SPSB1 + Akt-Myr-treated cells, #$p < 0.05$, ##$p < 0.01$, ###$p < 0.001$. n = 3 biologically independent experiments; data are presented as Mean ± standard deviation.

Figure S17. (A) Primary myoblasts were transduced by control GFP, SPSB1, Akt-Myr, respectively, or co-transduced by Akt-Myr and SPSB1 retrovirus and differentiated for 3 days. OP-puro labelling was performed for 1 h. Red fluorescence corresponds to de

novo synthesized polypeptides. (B) Primary myoblasts were transduced by control GFP, SPSB1, Myogenin, respectively, or co-transduced by Myogenin and SPSB1 retrovirus and differentiated for 3 days. OP-puro labelling was performed for 1 h. Red fluorescence corresponds to de novo synthesized polypeptides.

Supplementary Figure 18. Eight-week-old male B6(C)/Rj-Tyr$^{c/c}$ mice were injected with 1*10^{12} vector genomes (vg) of AAV9 expressing shRNA_Spsb1 or control_shRNA. After 6 weeks mice were subjected to CLP or sham surgery. Analyses were performed 96h after surgery (sham: control_shRNA, n=6, shRNA_Spsb1: n=6; CLP: control_shRNA, n=6, shRNA_Spsb1: n=6). (A) Experimental design. (B) qRT-PCR analysis of *Spsb1* expression in *tibialis anterior* and *gastrocnemius/plantaris* muscle. *p<0.05, **p<0.01; n.s. denotes not significant. Data are presented as Mean ± standard deviation. (C) Body weight and weights of *tibialis anterior* and *gastrocnemius/plantaris* muscle of CLP operated mice normalized to tibia length and expressed as relative change compared to sham operated mice. Data are presented as Mean ± standard deviation. *p<0.05, ***p<0.001. (D) Frequency distribution histograms of myofiber cross sectional area (MCSA) of Sham- and CLP-treated control_shRNA and shRNA_Spsb1 mice of histological cross sections from *tibialis anterior* muscle. ***p<0.001. (E, F) qRT-PCR analysis of *Trim63* and *Fbxo32* expression in *tibialis anterior* (E) and *gastrocnemius/plantaris* muscle (F). *p<0.05, **p<0.01, ***p<0.001. Data are presented as Mean ± standard deviation. n.s. denotes not significant.

Figure S19. Schematic model of SPSB1-mediated inhibition of myogenesis by

downregulating TGF-β-Akt-Myogenin signaling. Under physiological conditions, TGF-β binds to TβRI and TβRII complex and activates the non-canonical Akt and the canonical Smad pathway. TGF-β promotes myogenesis via the non-canonical TβRII-Akt-Myogenin pathway. Akt acts as a central regulator of transcription and translation, and increases protein synthesis, which is required for initiating myoblast fusion. Additionally, Akt induces expression of Myogenin/*Myog*, which in turn increases the expression of Myomaker/*Mymk*, Myomerger/*Mymx* as well as myosin heavy chain (MyHC) *Myh1, Myh3* and *Myh7*. An increase in proinflammatory cytokines, e.g., tumor necrosis factor (TNF), interleukin 1β (IL-1β) and IL6, during sepsis cause an increase in *Spsb1* expression by an activation of NF-κB and gp130/JAK2/STAT3 signaling, respectively, in myocytes. SPSB1 targets TβRII and inhibits the TβRII-Akt-Myogenin pathway decreasing protein synthesis, myogenic fusion and differentiation contributing to impaired myogenic differentiation. Created with BioRender.com.

References

1. Hahn A, Kny M, Pablo-Tortola C, Todiras M, Willenbrock M, Schmidt S, et al. Serum amyloid A1 mediates myotube atrophy via Toll-like receptors. J Cachexia Sarcopenia Muscle. 2020;11:103-19.

2. Zanders L, Kny M, Hahn A, Schmidt S, Wundersitz S, Todiras M, et al. Sepsis induces interleukin 6, gp130/JAK2/STAT3, and muscle wasting. J Cachexia Sarcopenia Muscle. 2022;13:713-27.

3. Zhu X, Kny M, Schmidt F, Hahn A, Wollersheim T, Kleber C, et al. Secreted

Frizzled-Related Protein 2 and Inflammation-Induced Skeletal Muscle Atrophy. Crit Care Med. 2017;45:e169-e83.

4. Boden D, Pusch O, Silbermann R, Lee F, Tucker L, Ramratnam B. Enhanced gene silencing of HIV-1 specific siRNA using microRNA designed hairpins. Nucleic Acids Res. 2004;32:1154-8.

5. Jungmann A, Leuchs B, Rommelaere J, Katus HA, Muller OJ. Protocol for Efficient Generation and Characterization of Adeno-Associated Viral Vectors. Hum Gene Ther Methods. 2017;28:235-46.

6. Fielitz J, Kim MS, Shelton JM, Latif S, Spencer JA, Glass DJ, et al. Myosin accumulation and striated muscle myopathy result from the loss of muscle RING finger 1 and 3. J Clin Invest. 2007;117:2486-95.

7. Kim MS, Fielitz J, McAnally J, Shelton JM, Lemon DD, McKinsey TA, et al. Protein kinase D1 stimulates MEF2 activity in skeletal muscle and enhances muscle performance. Mol Cell Biol. 2008;28:3600-9.

8. Huang N, Kny M, Riediger F, Busch K, Schmidt S, Luft FC, et al. Deletion of Nlrp3 protects from inflammation-induced skeletal muscle atrophy. Intensive Care Med Exp. 2017;5:3.

9. Schmidt F, Kny M, Zhu X, Wollersheim T, Persicke K, Langhans C, et al. The E3 ubiquitin ligase TRIM62 and inflammation-induced skeletal muscle atrophy. Crit Care. 2014;18:545.

10. Wollersheim T, Woehlecke J, Krebs M, Hamati J, Lodka D, Luther-Schroeder A, et al. Dynamics of myosin degradation in intensive care unit-acquired weakness during

severe critical illness. Intensive Care Med. 2014;40:528-38.

11. Lodka D, Pahuja A, Geers-Knorr C, Scheibe RJ, Nowak M, Hamati J, et al. Muscle RING-finger 2 and 3 maintain striated-muscle structure and function. J Cachexia Sarcopenia Muscle. 2016;7:165-80.

12. Benedetti A, Cera G, De Meo D, Villani C, Bouche M, Lozanoska-Ochser B. A Simple Method for the Isolation and in vitro Expansion of Highly Pure Mouse and Human Satellite Cells. Bio Protoc. 2021;11:e4238.

13. Aoki M, Batista O, Bellacosa A, Tsichlis P, Vogt PK. The akt kinase: molecular determinants of oncogenicity. Proc Natl Acad Sci U S A. 1998;95:14950-5.

14. Du Bois P, Pablo Tortola C, Lodka D, Kny M, Schmidt F, Song K, et al. Angiotensin II Induces Skeletal Muscle Atrophy by Activating TFEB-Mediated MuRF1 Expression. Circ Res. 2015;117:424-36.

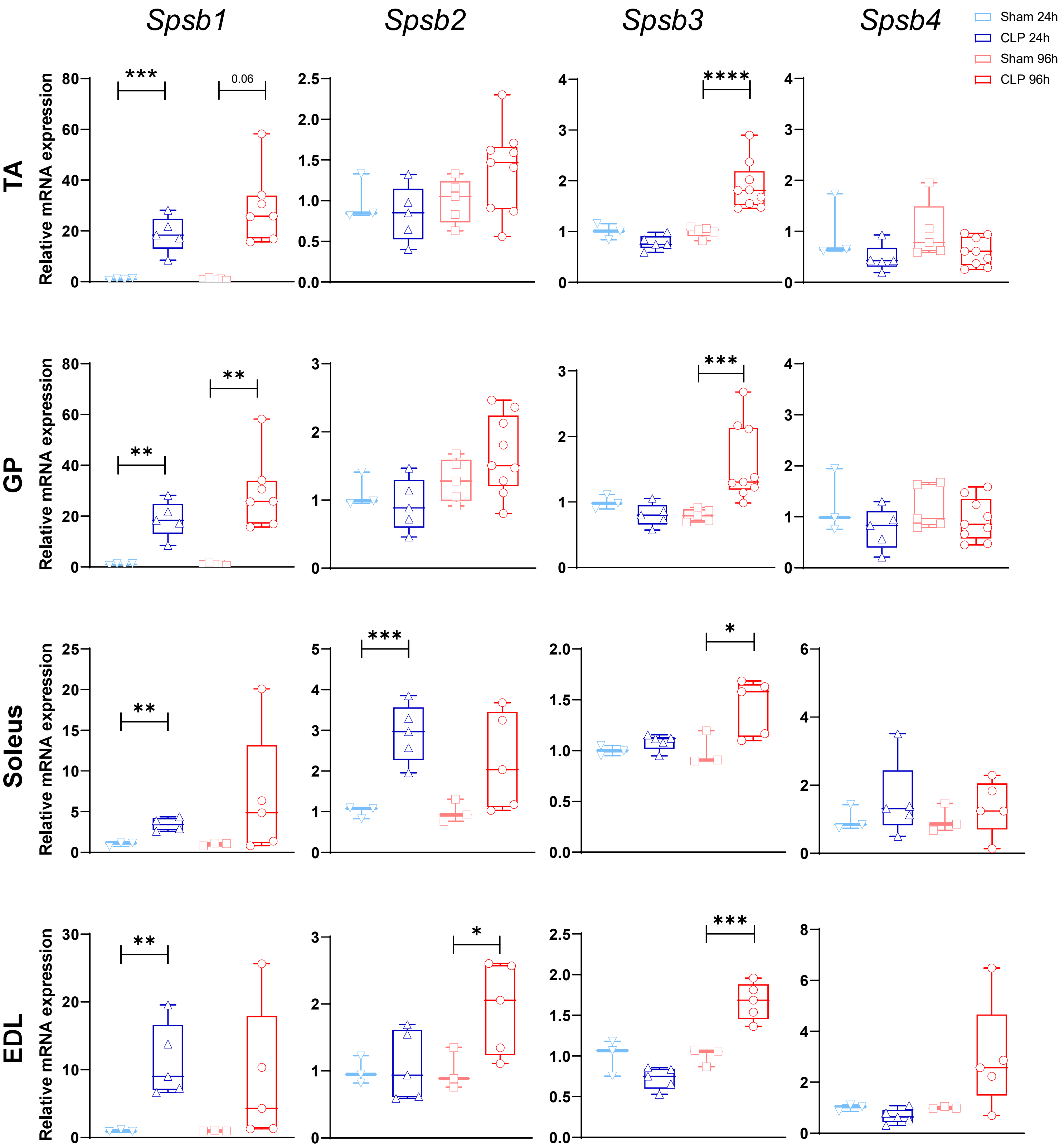

Spsb1
Spsb2
Spsb3
Spsb4
Sham 24h
CLP 24h
Sham 96h
CLP 96h
TA
GP
Soleus
EDL
Relative mRNA expression
***
0.06
****
**
***
*

Figure S1

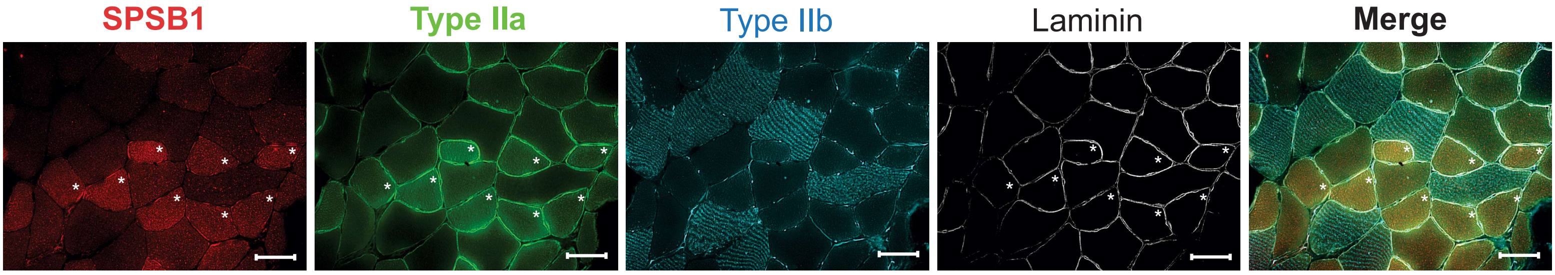

Figure S2

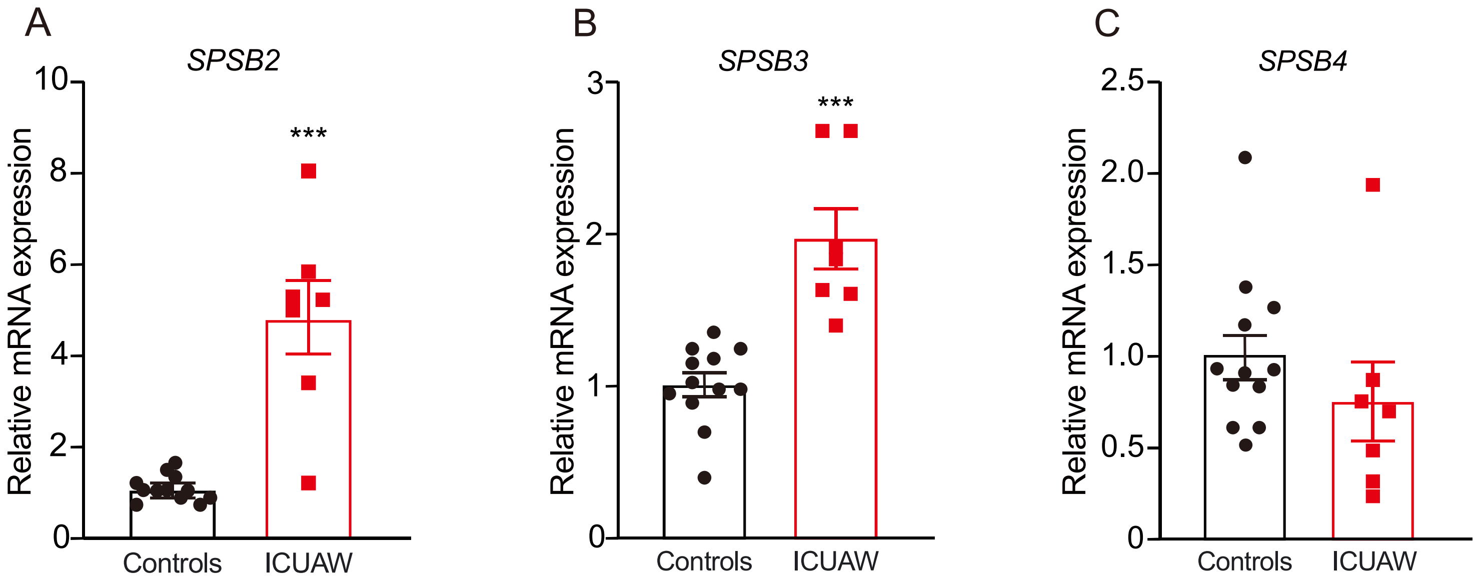

A
SPSB2
***
Relative mRNA expression
10
8
6
4
2
0
Controls
ICUAW
B
SPSB3
***
Relative mRNA expression
3
2
1
0
Controls
ICUAW
C
SPSB4
Relative mRNA expression
2.5
2.0
1.5
1.0
0.5
0
Controls
ICUAW

Figure S3

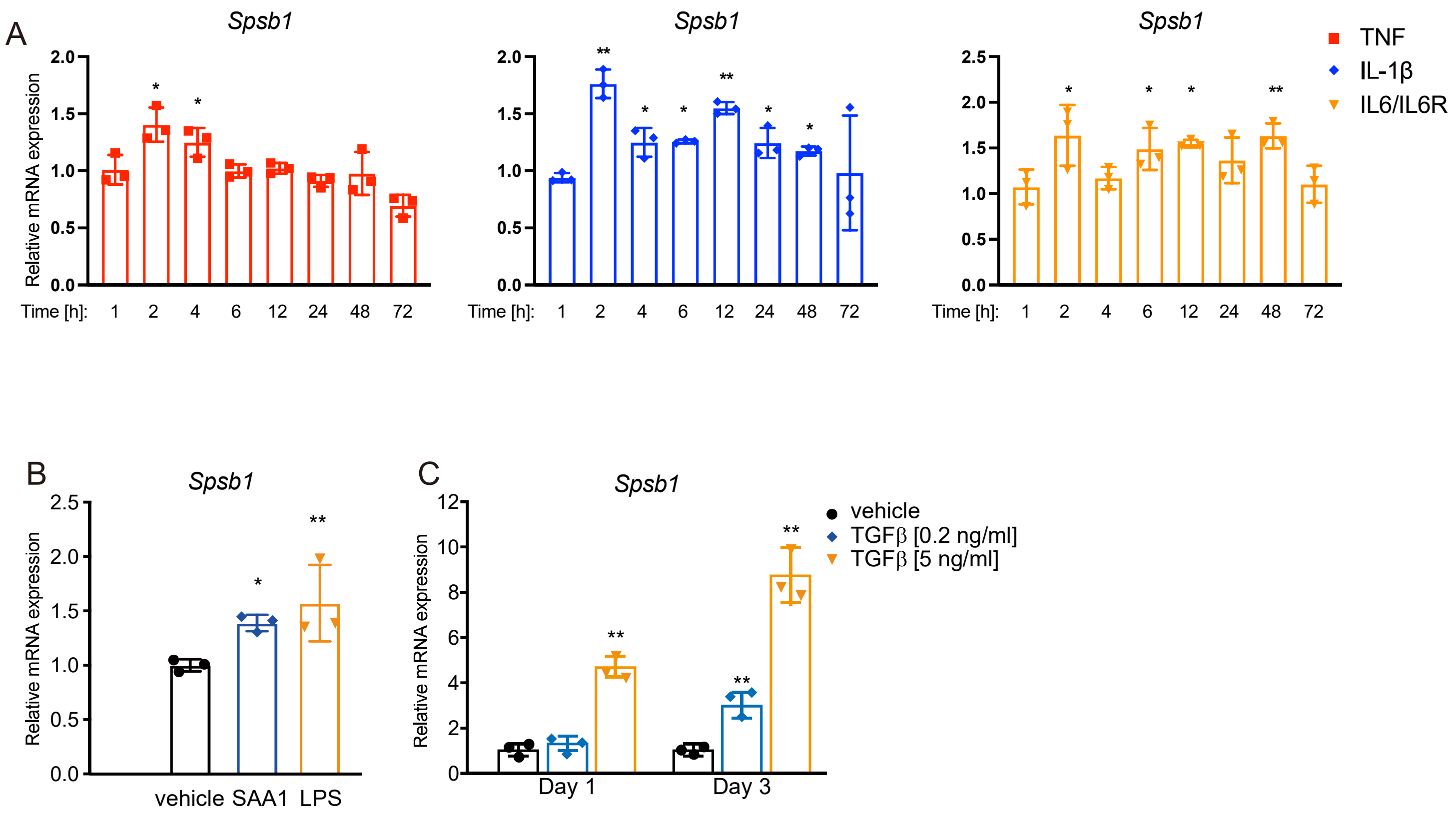
A
Spsb1
Spsb1
Spsb1
TNF
IL-1β
IL6/IL6R
Relative mRNA expression
Time [h]: 1 2 4 6 12 24 48 72
B
Spsb1
Relative mRNA expression
vehicle SAA1 LPS
C
Spsb1
Relative mRNA expression
vehicle
TGFβ [0.2 ng/ml]
TGFβ [5 ng/ml]
Day 1
Day 3

Figure S4

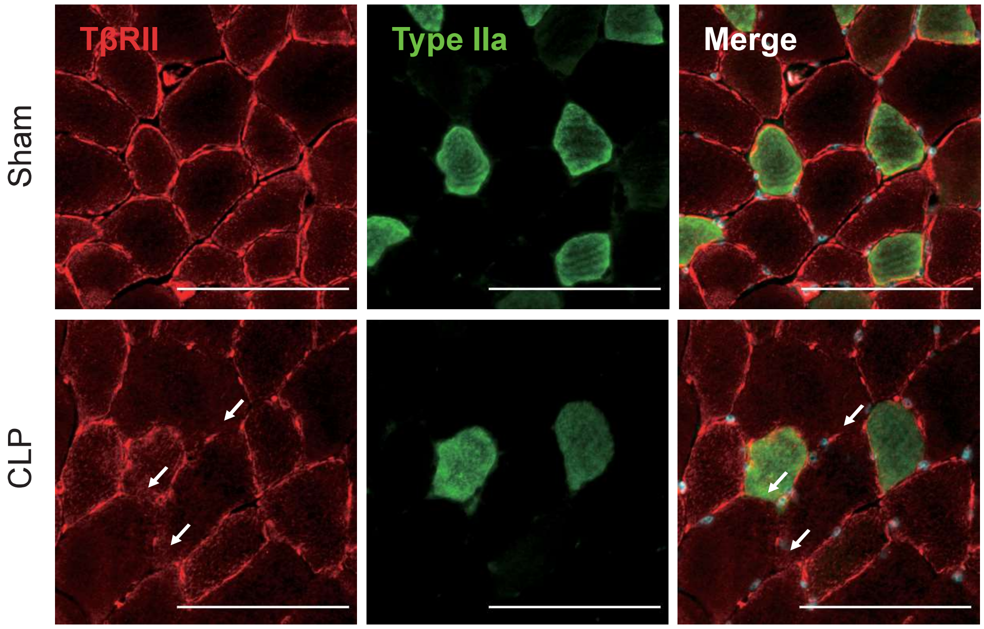

Figure S5

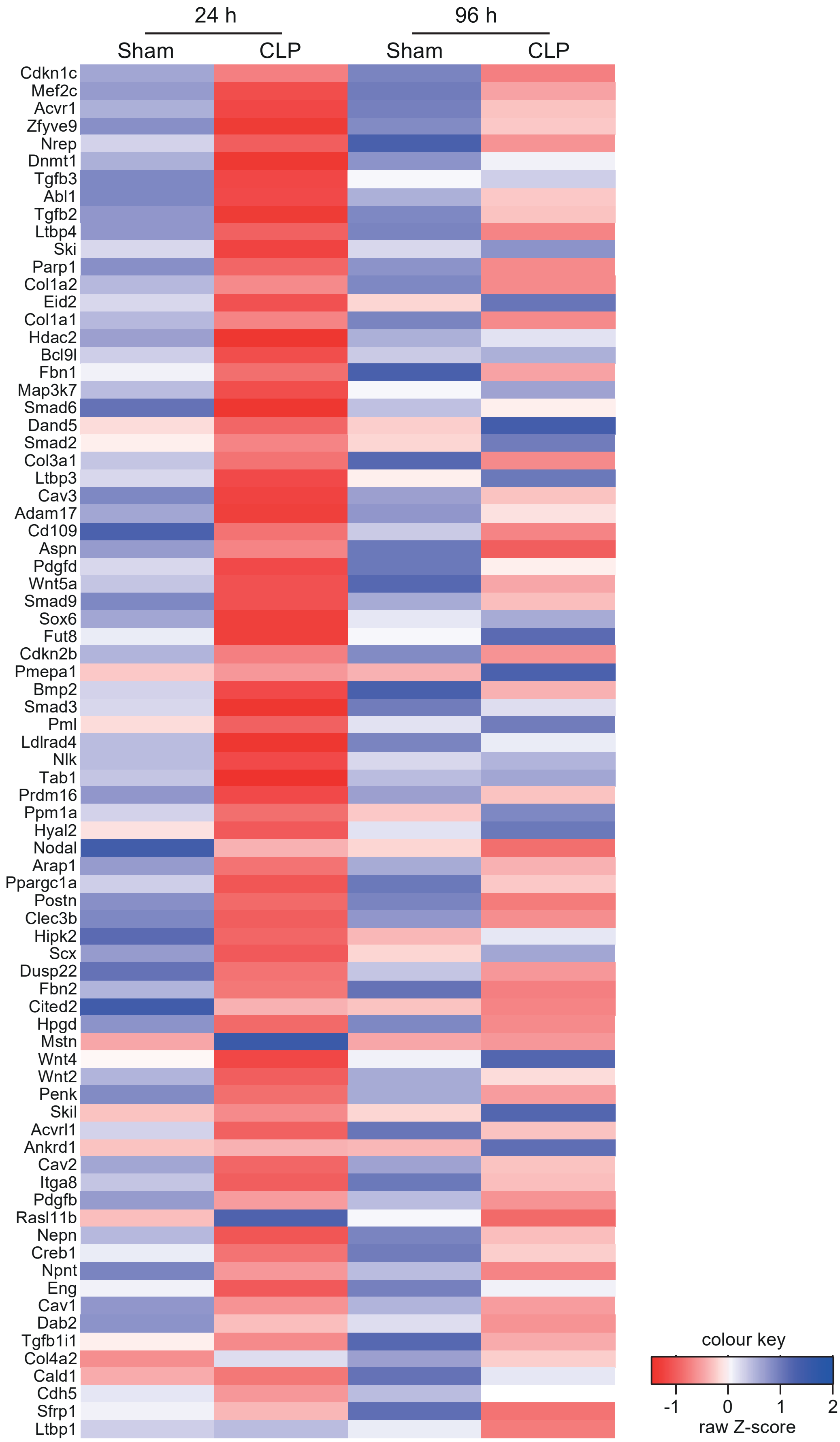

24 h
96 h
Sham
CLP
Sham
CLP
Cdkn1c
Mef2c
Acvr1
Zfyve9
Nrep
Dnmt1
Tgfb3
Abl1
Tgfb2
Ltbp4
Ski
Parp1
Col1a2
Eid2
Col1a1
Hdac2
Bcl9l
Fbn1
Map3k7
Smad6
Dand5
Smad2
Col3a1
Ltbp3
Cav3
Adam17
Cd109
Aspn
Pdgfd
Wnt5a
Smad9
Sox6
Fut8
Cdkn2b
Pmepa1
Bmp2
Smad3
Pml
Ldlrad4
Nlk
Tab1
Prdm16
Ppm1a
Hyal2
Nodal
Arap1
Ppargc1a
Postn
Clec3b
Hipk2
Scx
Dusp22
Fbn2
Cited2
Hpgd
Mstn
Wnt4
Wnt2
Penk
Skil
Acvrl1
Ankrd1
Cav2
Itga8
Pdgfb
Rasl11b
Nepn
Creb1
Npnt
Eng
Cav1
Dab2
Tgfb1i1
Col4a2
Cald1
Cdh5
Sfrp1
Ltbp1
colour key
-1
0
1
2
raw Z-score

Figure S6

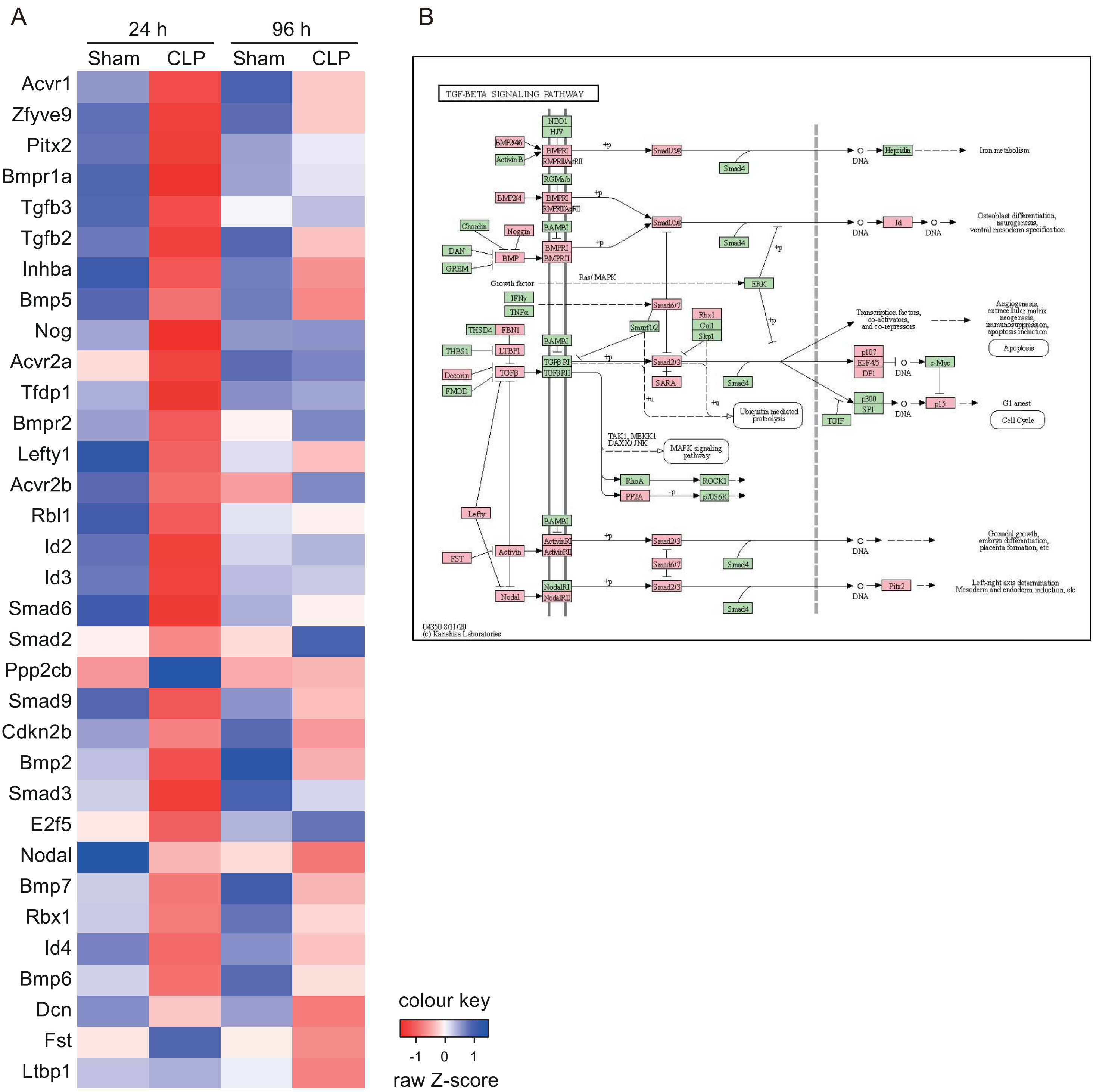
A
24 h
96 h
Sham
CLP
Sham
CLP
Acvr1
Zfyve9
Pitx2
Bmpr1a
Tgfb3
Tgfb2
Inhba
Bmp5
Nog
Acvr2a
Tfdp1
Bmpr2
Lefty1
Acvr2b
Rbl1
Id2
Id3
Smad6
Smad2
Ppp2cb
Smad9
Cdkn2b
Bmp2
Smad3
E2f5
Nodal
Bmp7
Rbx1
Id4
Bmp6
Dcn
Fst
Ltbp1
B
TGF-BETA SIGNALING PATHWAY
Iron metabolism
Osteoblast differentiation, neurogenesis, ventral mesoderm specification
Angiogenesis, extracellular matrix neogenesis, immunosuppression, apoptosis induction
Apoptosis
G1 arrest
Cell Cycle
Gonadal growth, embryo differentiation, placenta formation, etc
Left-right axis determination Mesoderm and endoderm induction, etc
MAPK signaling pathway
Ubiquitin mediated proteolysis
04350 8/11/20
(c) Kanehisa Laboratories
colour key
-1
0
1
raw Z-score

Figure S7

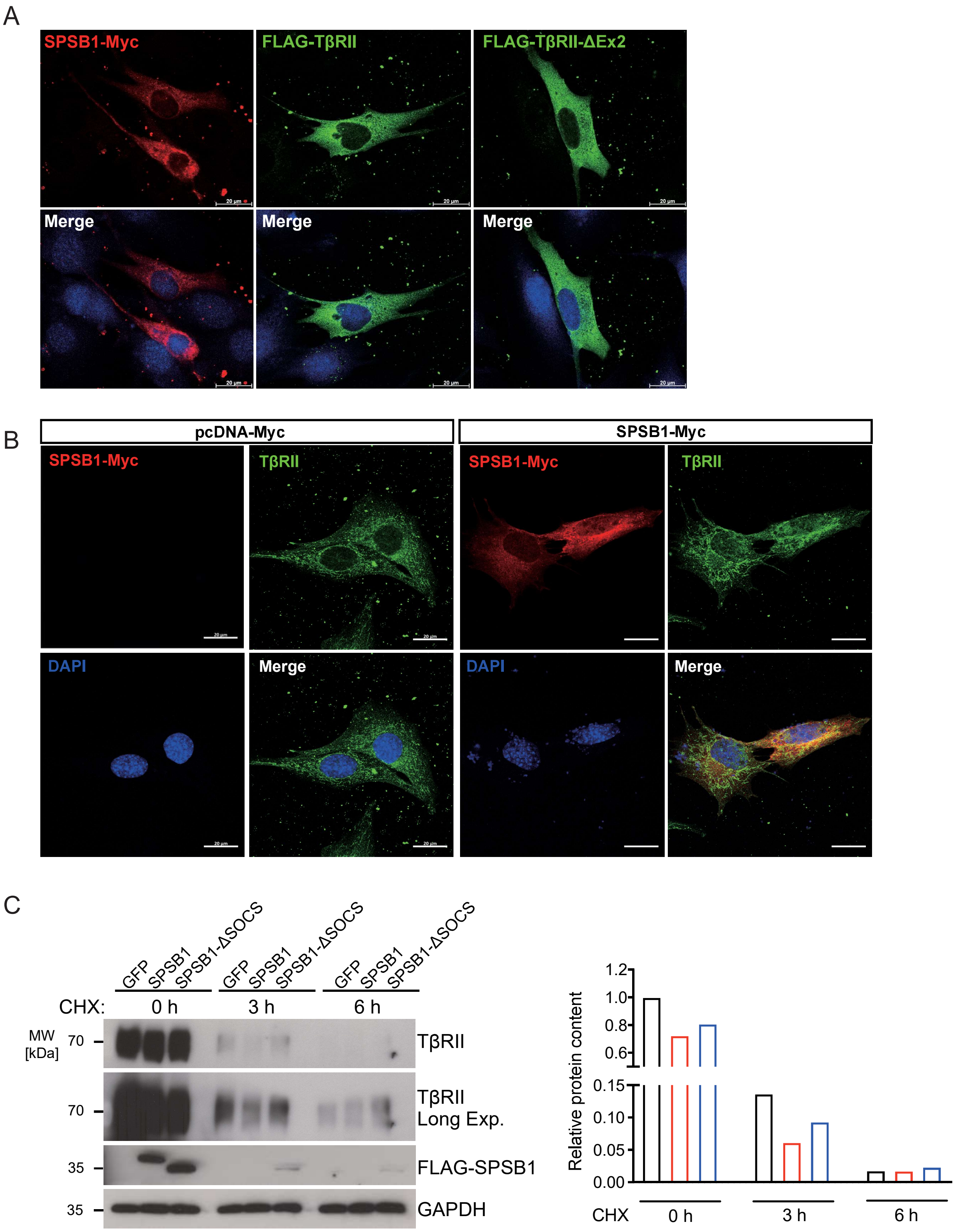
A
SPSB1-Myc
FLAG-TβRII
FLAG-TβRII-ΔEx2
Merge
Merge
Merge
B
pcDNA-Myc
SPSB1-Myc
SPSB1-Myc
TβRII
SPSB1-Myc
TβRII
DAPI
Merge
DAPI
Merge
C
GFP
SPSB1
SPSB1-ΔSOCS
CHX:
0 h
3 h
6 h
MW [kDa]
70
70
35
35
TβRII
TβRII Long Exp.
FLAG-SPSB1
GAPDH
Relative protein content
1.2
1.0
0.8
0.6
0.15
0.10
0.05
0.00
CHX
0 h
3 h
6 h

Figure S8

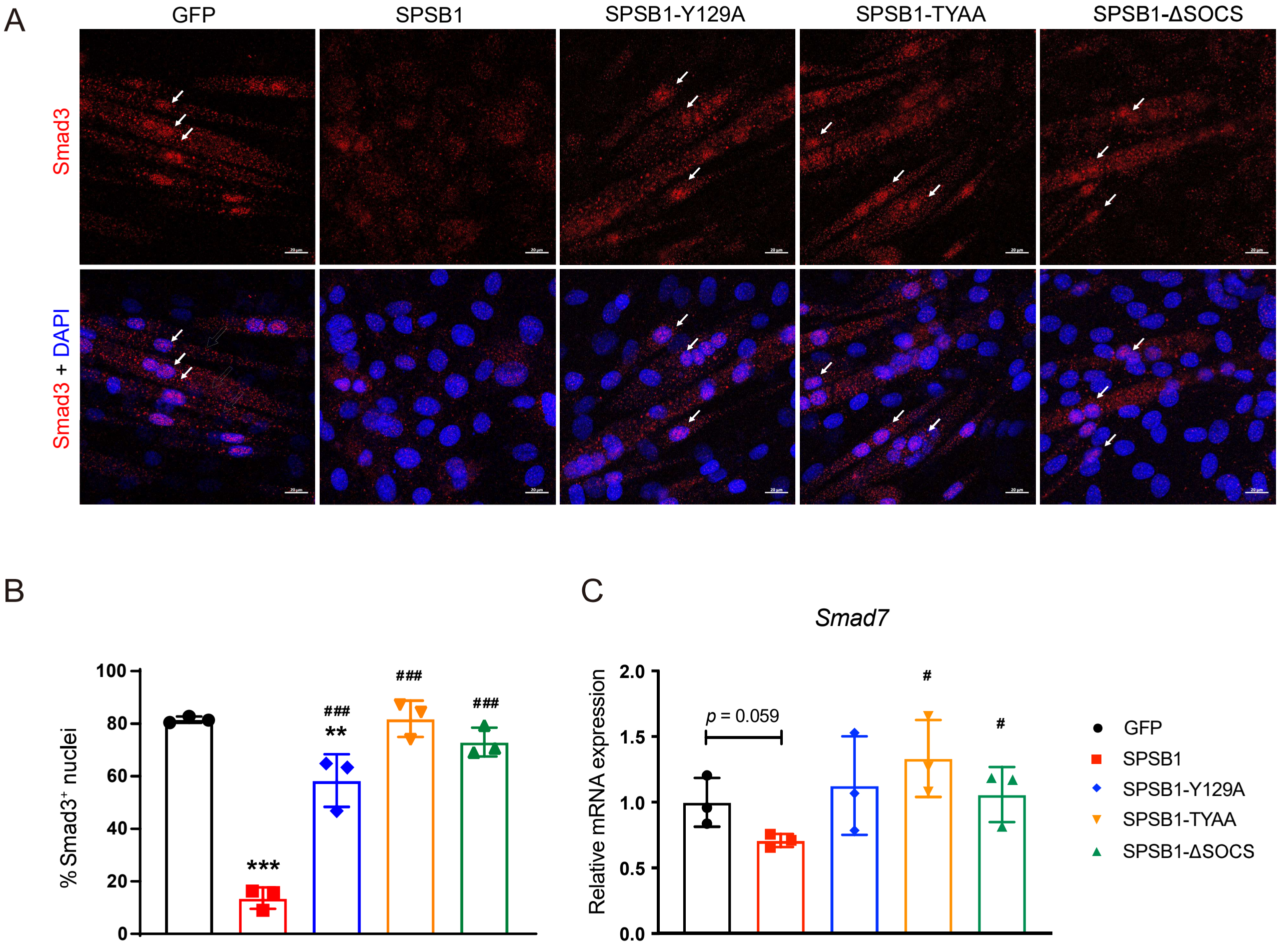
A
GFP
SPSB1
SPSB1-Y129A
SPSB1-TYAA
SPSB1-ΔSOCS
Smad3
Smad3 + DAPI
B
% Smad3⁺ nuclei
100
80
60
40
20
0
***
**
###
###
###
C
Smad7
Relative mRNA expression
2.0
1.5
1.0
0.5
0.0
p = 0.059
#
#
GFP
SPSB1
SPSB1-Y129A
SPSB1-TYAA
SPSB1-ΔSOCS

Figure S9

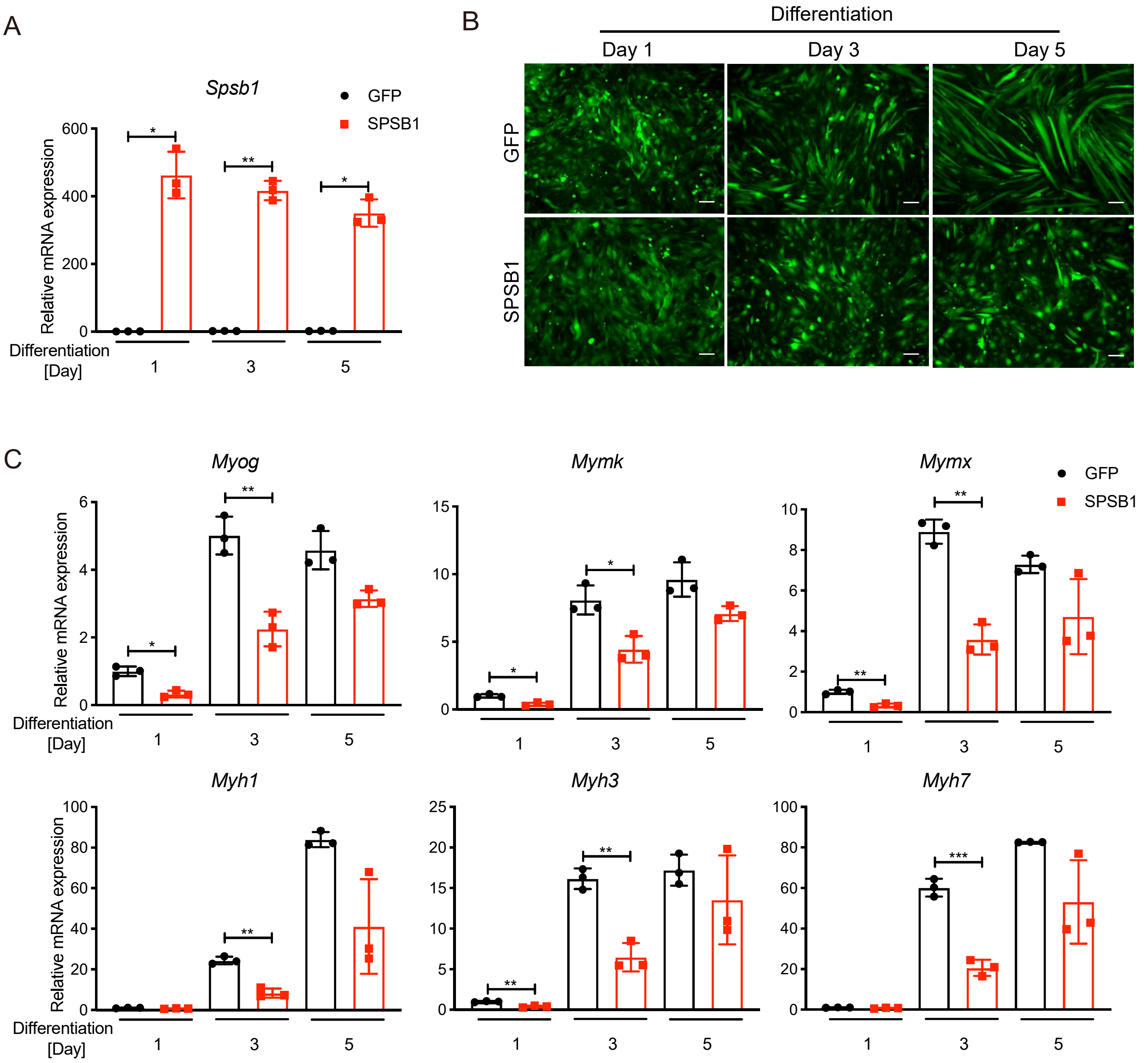

A
Spsb1
GFP
SPSB1
Relative mRNA expression
600
400
200
0
Differentiation
[Day]
1
3
5
B
Differentiation
Day 1
Day 3
Day 5
GFP
SPSB1
C
Myog
Mymk
Mymx
Myh1
Myh3
Myh7

Figure S10

A

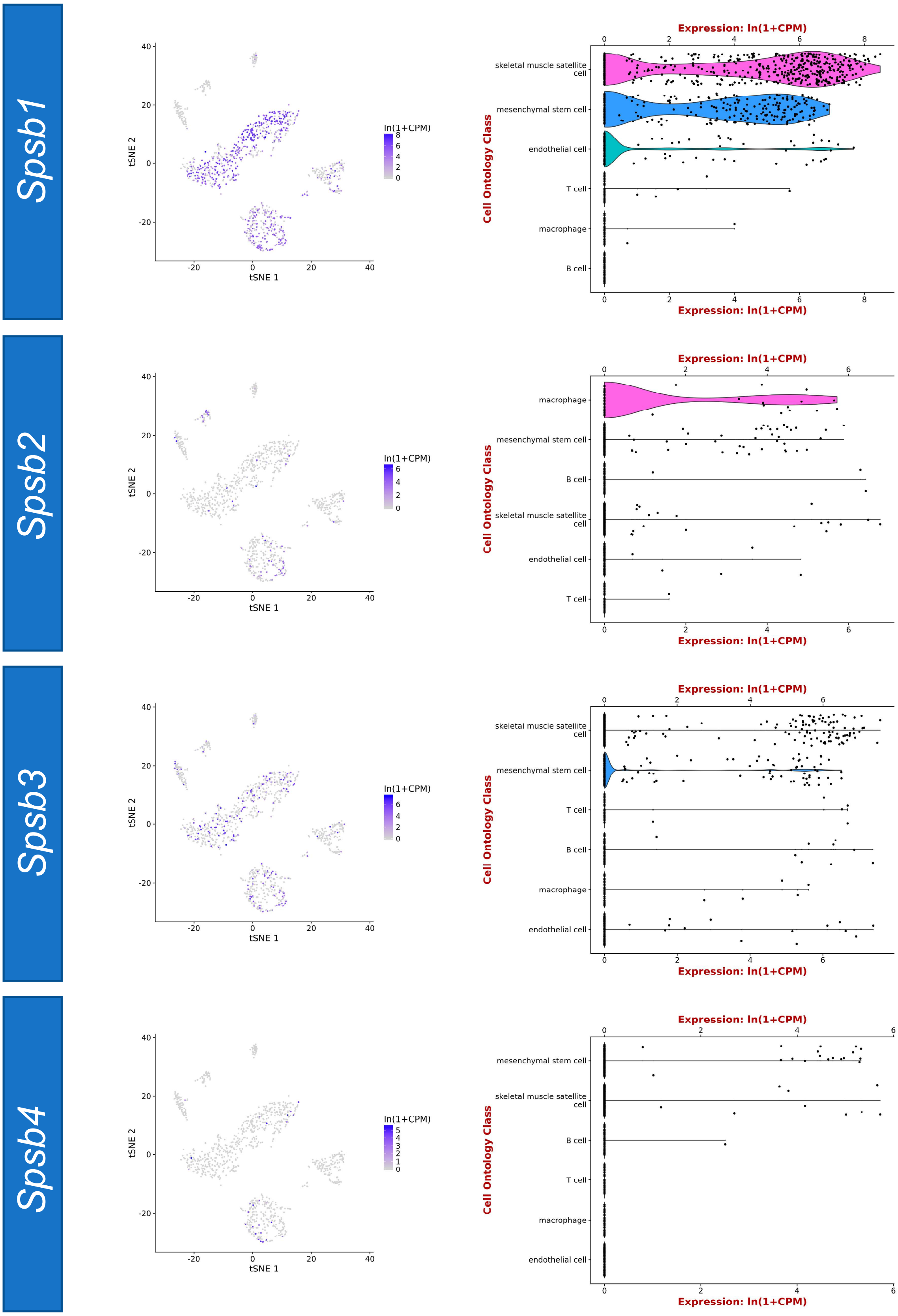

Tissue	Mean	Median	Median > 0	# cells	% 0	% > 0
skeletal muscle satellite cell	3.89	4.75	5.89	540	26.85	73.15
mesenchymal stem cell	2.96	3.45	4.68	258	31.40	68.60
endothelial cell	1.15	0.00	3.84	141	73.05	26.95
T cell	0.39	0.00	2.26	35	85.71	14.29
macrophage	0.10	0.00	2.35	45	95.56	4.44
B cell	0.00	0.00	NA	71	100.00	0.00

Tissue	Mean	Median	Median > 0	# cells	% 0	% > 0
macrophage	1.28	0	4.43	45	68.89	31.11
mesenchymal stem cell	0.53	0	3.86	258	84.50	15.50
B cell	0.20	0	6.29	71	95.77	4.23
skeletal muscle satellite cell	0.11	0	1.77	540	96.48	3.52
endothelial cell	0.10	0	2.87	141	96.45	3.55
T cell	0.05	0	1.59	35	97.14	2.86

Tissue	Mean	Median	Median > 0	# cells	% 0	% > 0
skeletal muscle satellite cell	1.05	0	5.77	540	79.63	20.37
mesenchymal stem cell	1.05	0	4.99	258	74.42	25.58
T cell	0.78	0	6.52	35	85.71	14.29
B cell	0.72	0	6.23	71	87.32	12.68
macrophage	0.50	0	4.88	45	88.89	11.11
endothelial cell	0.42	0	4.46	141	90.07	9.93

Tissue	Mean	Median	Median > 0	# cells	% 0	% > 0
mesenchymal stem cell	0.30	0	4.57	258	93.02	6.98
skeletal muscle satellite cell	0.07	0	4.16	540	98.33	1.67
B cell	0.04	0	2.51	71	98.59	1.41
T cell	0.00	0	NA	35	100.00	0.00
macrophage	0.00	0	NA	45	100.00	0.00
endothelial cell	0.00	0	NA	141	100.00	0.00

B

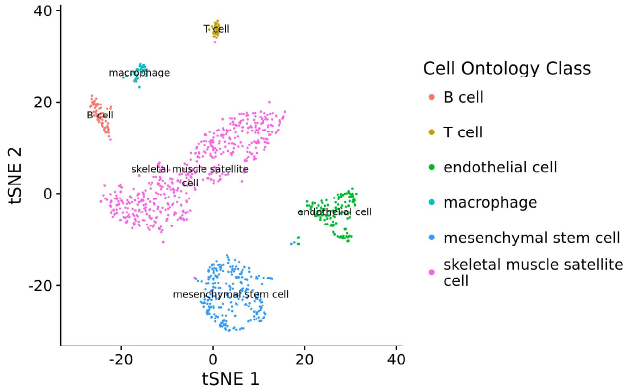

Figure S11

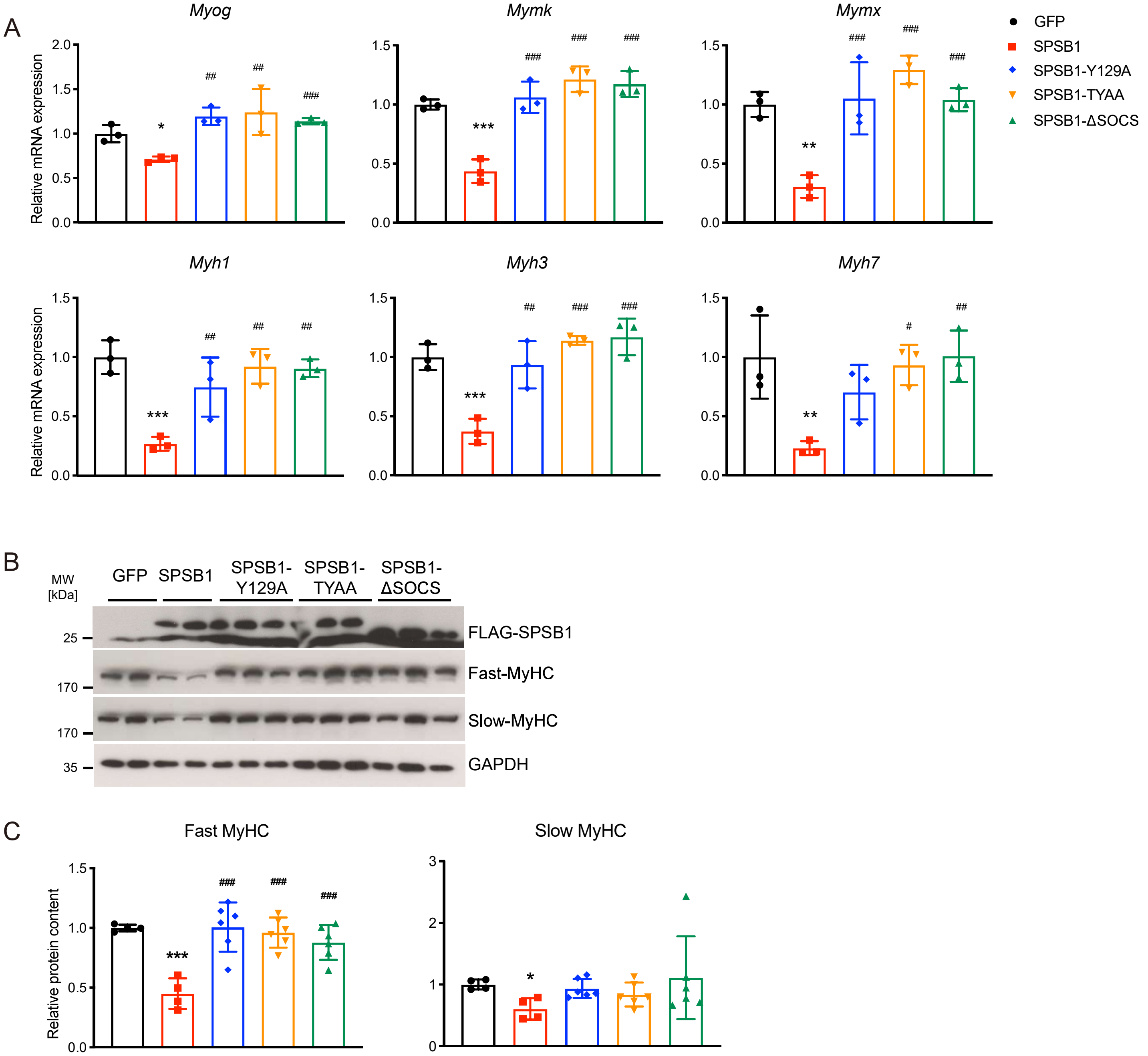
A
Myog
Mymk
Mymx
Myh1
Myh3
Myh7
Relative mRNA expression
GFP
SPSB1
SPSB1-Y129A
SPSB1-TYAA
SPSB1-ΔSOCS
B
MW [kDa]
GFP
SPSB1
SPSB1-Y129A
SPSB1-TYAA
SPSB1-ΔSOCS
FLAG-SPSB1
Fast-MyHC
Slow-MyHC
GAPDH
C
Fast MyHC
Slow MyHC
Relative protein content

Figure S12

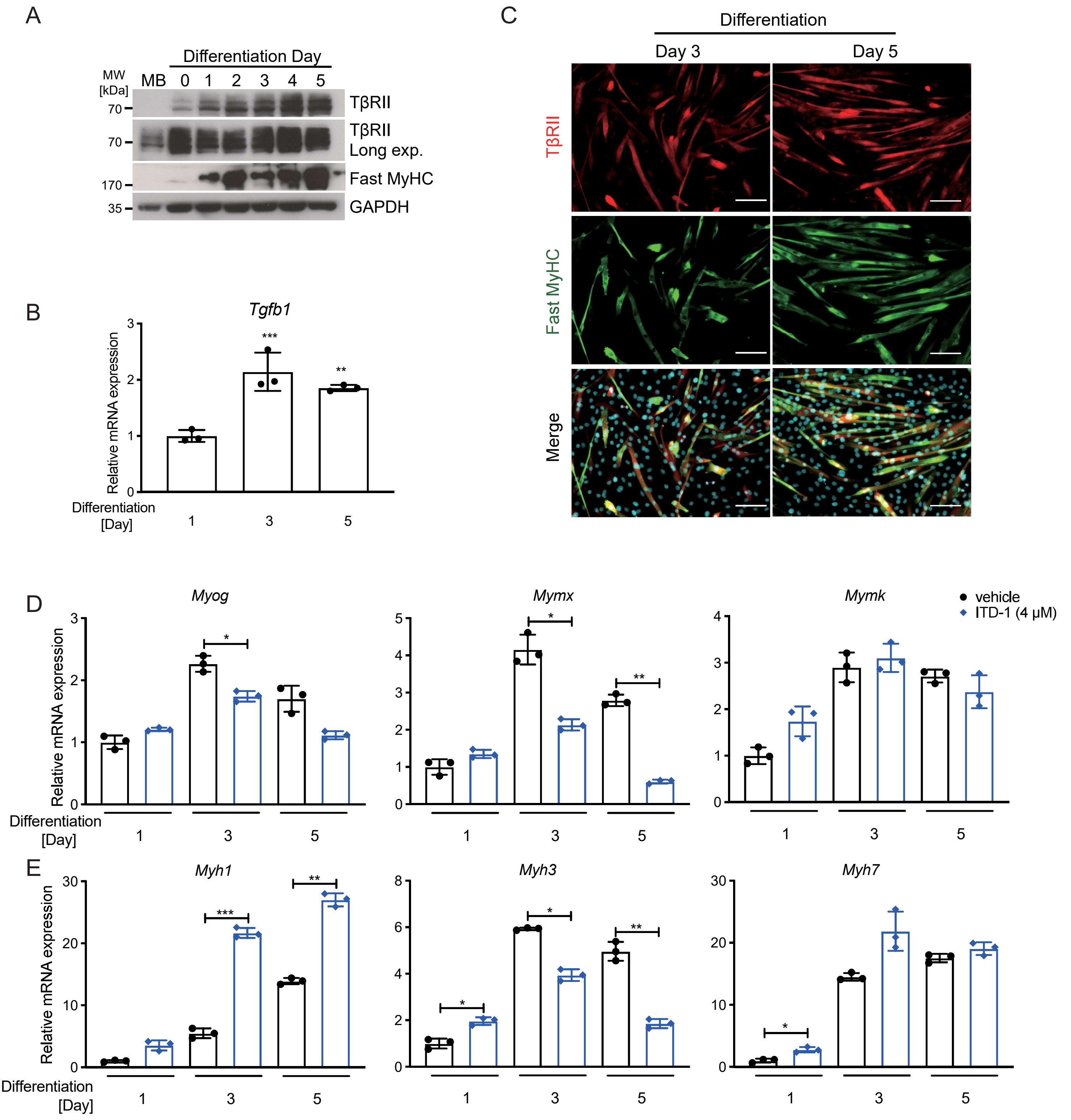
A
Differentiation Day
MW [kDa]
MB 0 1 2 3 4 5
70
TβRII
70
TβRII Long exp.
170
Fast MyHC
35
GAPDH
B
Tgfb1
Relative mRNA expression
***
**
Differentiation [Day]
1 3 5
C
Differentiation
Day 3
Day 5
TβRII
Fast MyHC
Merge
D
Myog
Mymx
Mymk
vehicle
ITD-1 (4 μM)
Relative mRNA expression
Differentiation [Day]
1 3 5
E
Myh1
Myh3
Myh7
Relative mRNA expression
Differentiation [Day]
1 3 5

Figure S13

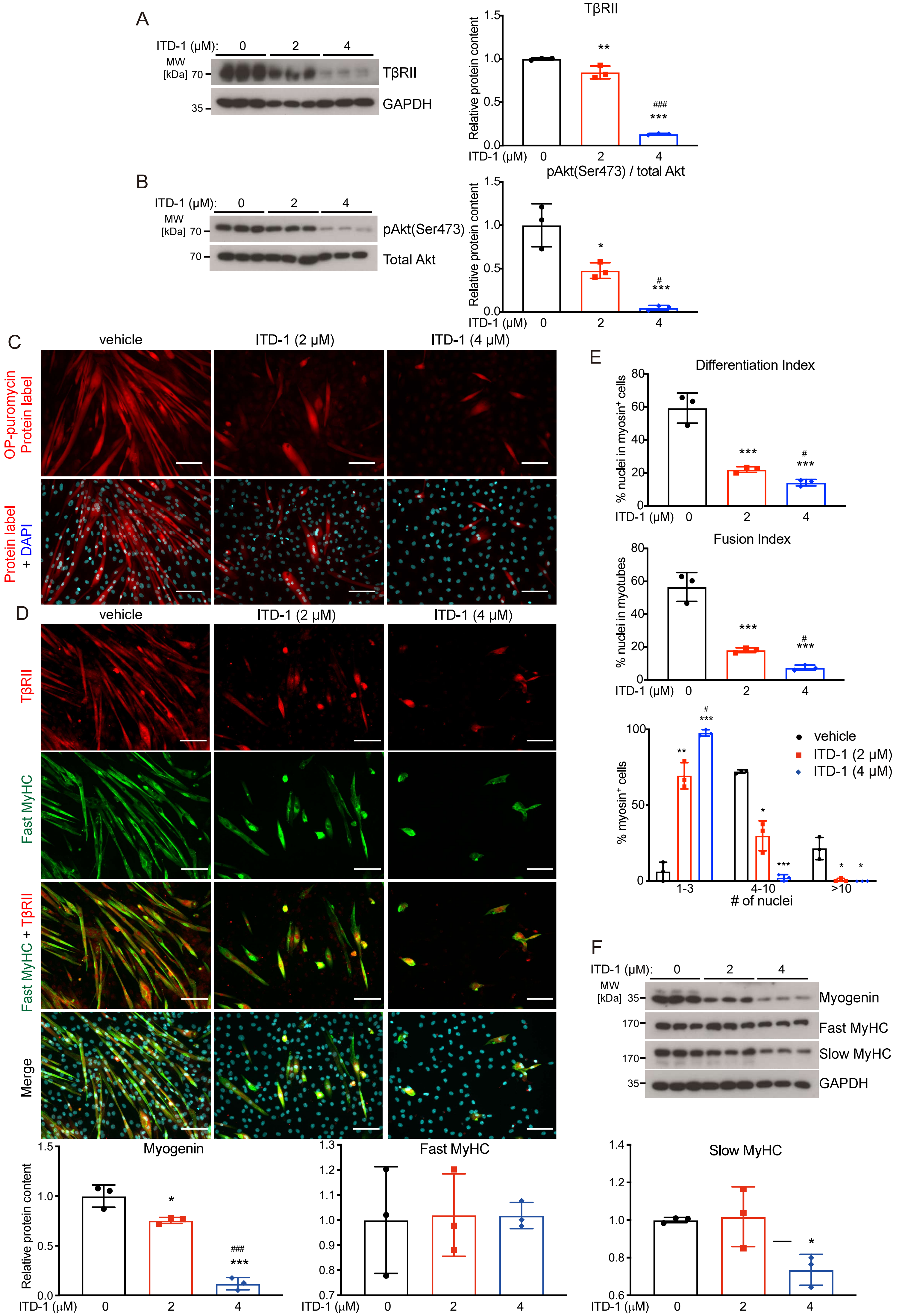
A
ITD-1 (μM): 0 2 4
MW [kDa] 70
TβRII
35
GAPDH
TβRII
Relative protein content
1.5
1.0
0.5
0.0
**
###
***
ITD-1 (μM) 0 2 4
B
ITD-1 (μM): 0 2 4
MW [kDa] 70
pAkt(Ser473)
70
Total Akt
pAkt(Ser473) / total Akt
Relative protein content
*
#
***
C
vehicle
ITD-1 (2 μM)
ITD-1 (4 μM)
OP-puromycin Protein label
Protein label + DAPI
D
TβRII
Fast MyHC
Fast MyHC + TβRII
Merge
E
Differentiation Index
% nuclei in myosin+ cells
80
60
40
20
0
***
#
***
ITD-1 (μM) 0 2 4
Fusion Index
% nuclei in myotubes
% myosin+ cells
100
50
vehicle
ITD-1 (2 μM)
ITD-1 (4 μM)
1-3
4-10
>10
# of nuclei
F
ITD-1 (μM): 0 2 4
MW [kDa] 35
Myogenin
170
Fast MyHC
170
Slow MyHC
35
GAPDH
Myogenin
Relative protein content
Fast MyHC
1.3
1.2
1.1
1.0
0.9
0.8
0.7
Slow MyHC
1.4
1.2
1.0
0.8
0.6

Figure S14

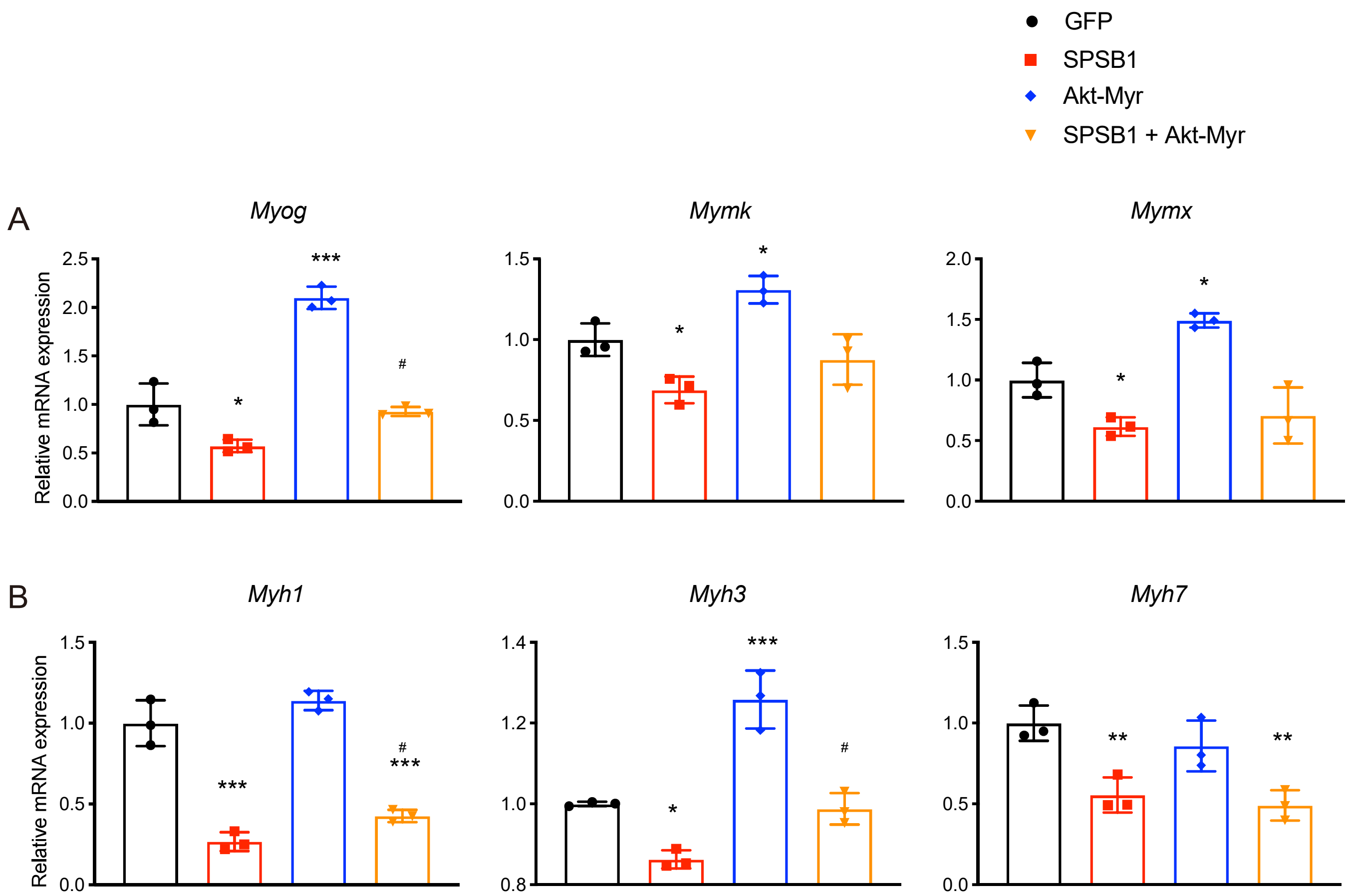
GFP
SPSB1
Akt-Myr
SPSB1 + Akt-Myr
A
Myog
Mymk
Mymx
Relative mRNA expression
B
Myh1
Myh3
Myh7

Figure S15

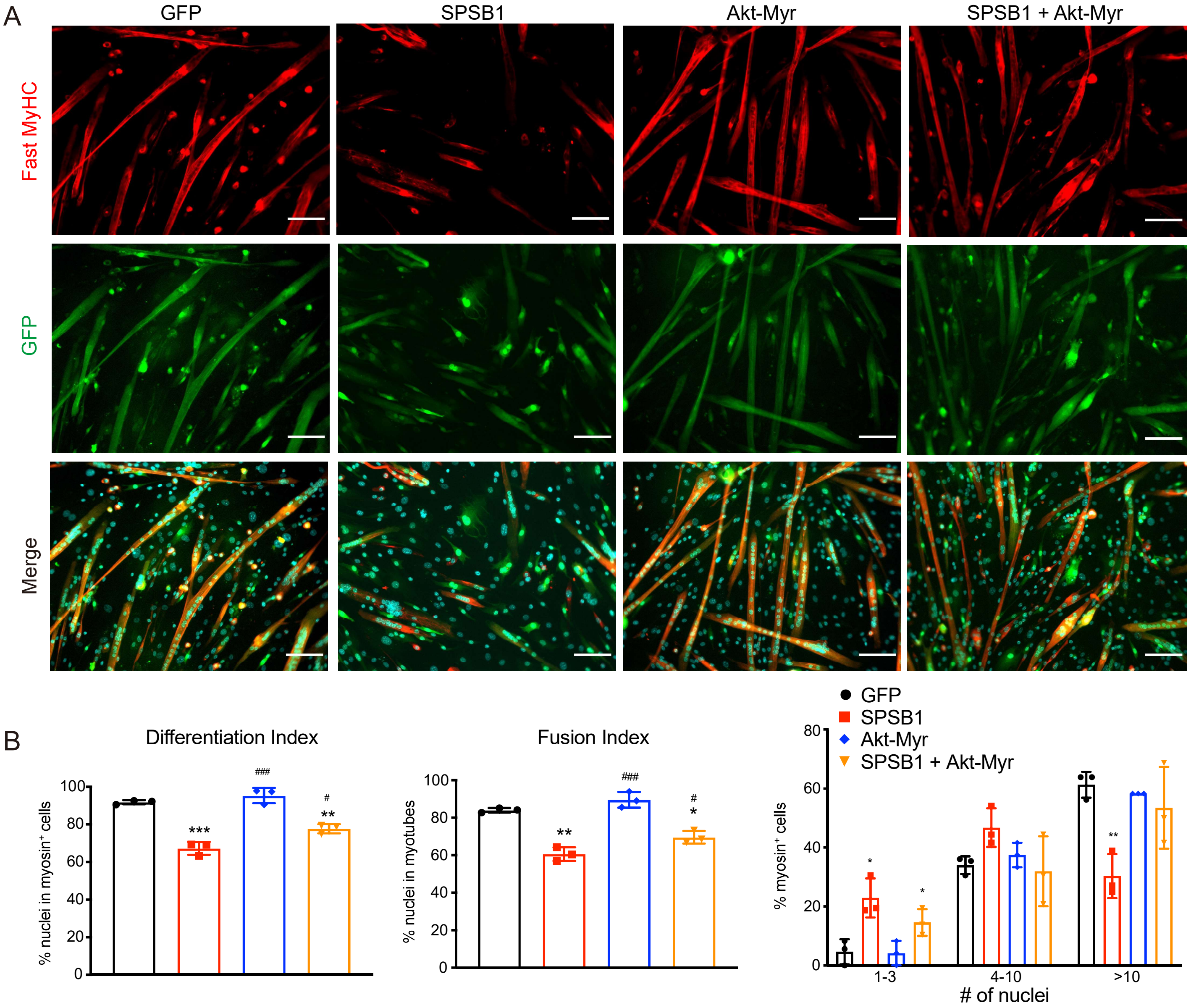

A
GFP
SPSB1
Akt-Myr
SPSB1 + Akt-Myr
Fast MyHC
GFP
Merge
B
Differentiation Index
% nuclei in myosin+ cells
***
###
#
**
Fusion Index
% nuclei in myotubes
**
###
#
*
GFP
SPSB1
Akt-Myr
SPSB1 + Akt-Myr
% myosin+ cells
1-3
4-10
>10
# of nuclei

Figure S16

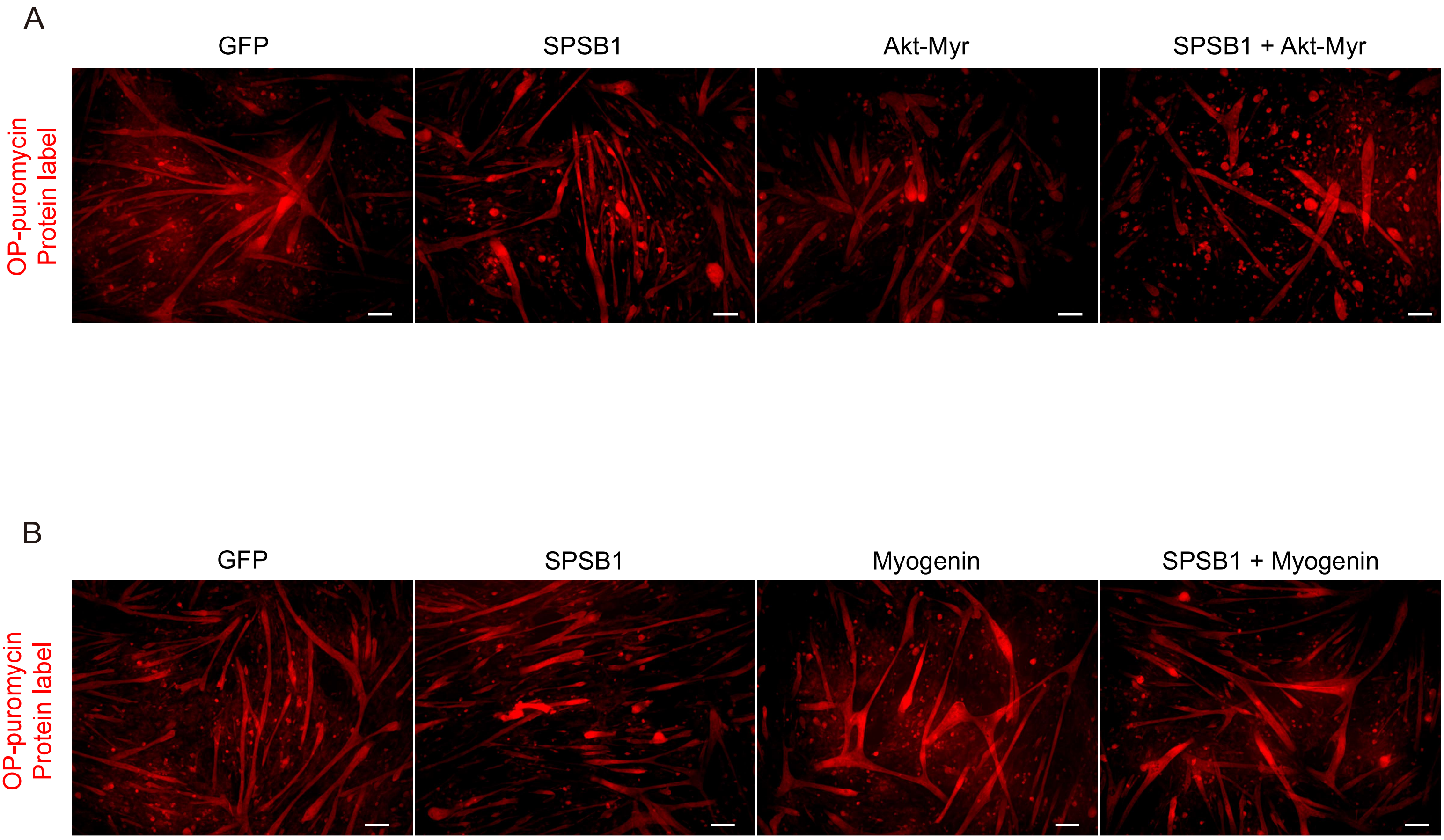
A
GFP
SPSB1
Akt-Myr
SPSB1 + Akt-Myr
OP-puromycin
Protein label
B
GFP
SPSB1
Myogenin
SPSB1 + Myogenin
OP-puromycin
Protein label

Figure S17

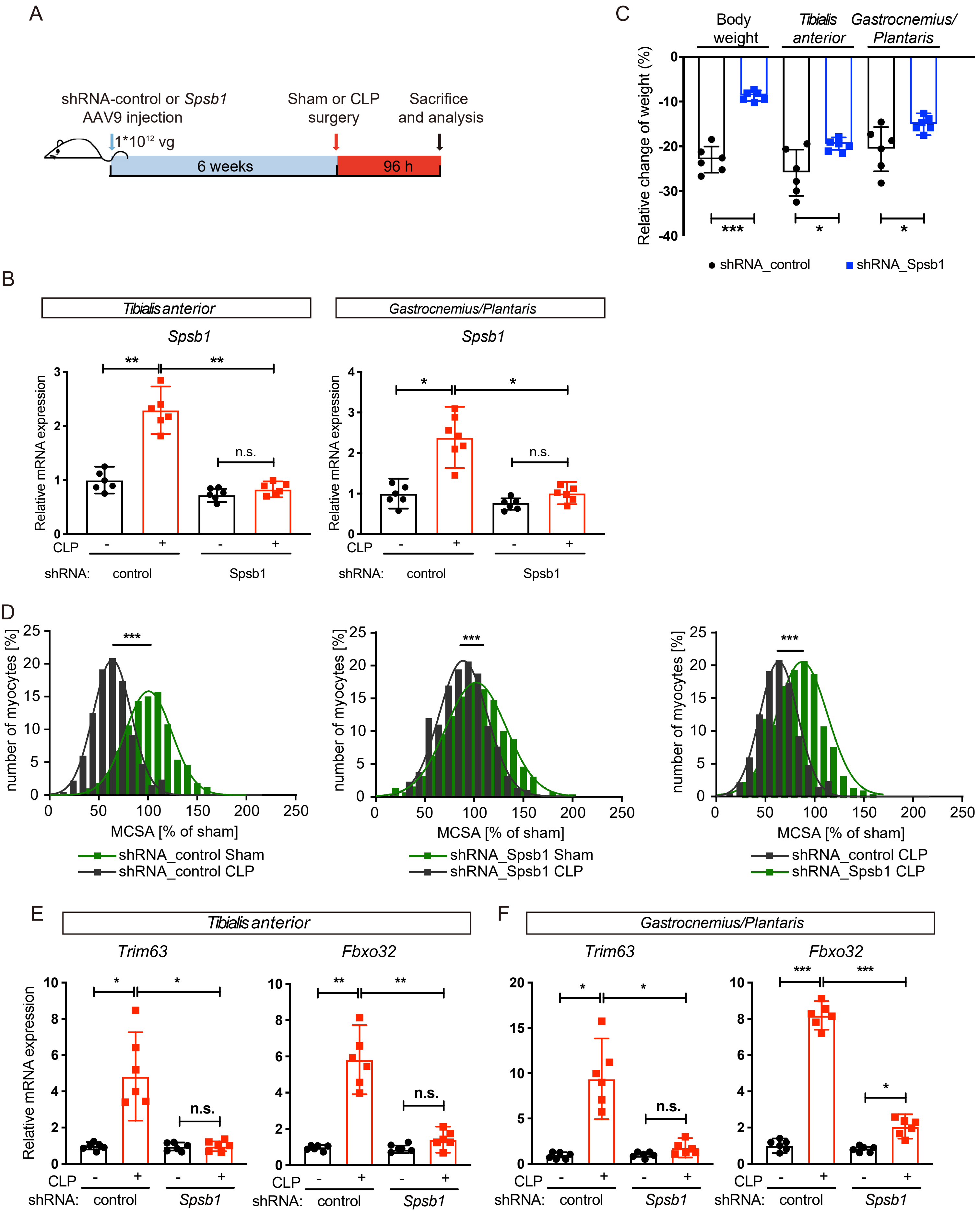
A
shRNA-control or Spsb1 AAV9 injection
1*10^12 vg
Sham or CLP surgery
Sacrifice and analysis
6 weeks
96 h
C
Body weight
Tibialis anterior
Gastrocnemius/ Plantaris
Relative change of weight (%)
shRNA_control
shRNA_Spsb1
B
Tibialis anterior
Gastrocnemius/Plantaris
Spsb1
Relative mRNA expression
n.s.
CLP
shRNA: control Spsb1
D
number of myocytes [%]
MCSA [% of sham]
shRNA_control Sham
shRNA_control CLP
shRNA_Spsb1 Sham
shRNA_Spsb1 CLP
E
Tibialis anterior
F
Gastrocnemius/Plantaris
Trim63
Fbxo32
Relative mRNA expression
CLP
shRNA: control Spsb1

Figure S18

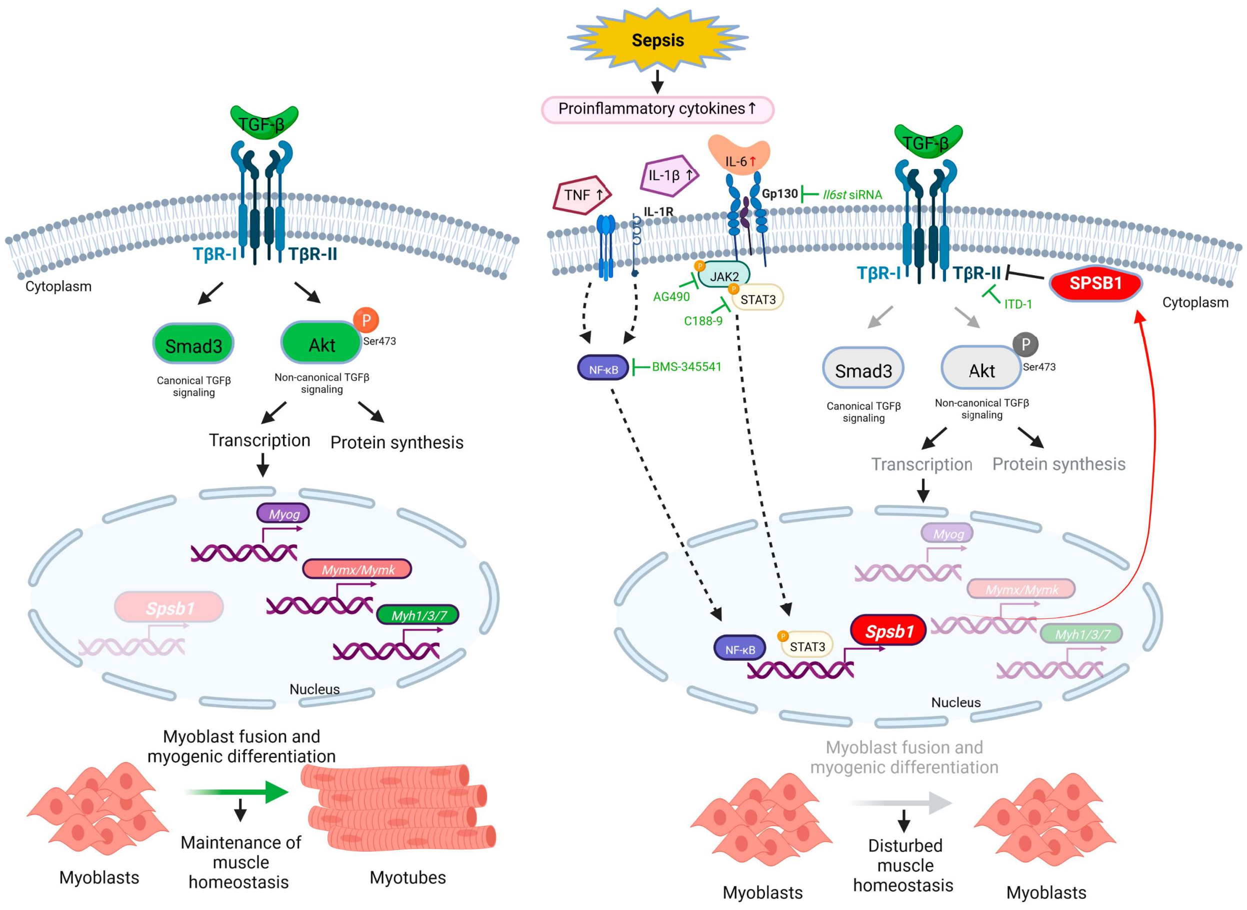
Sepsis
Proinflammatory cytokines↑
TGF-β
TβR-I
TβR-II
Cytoplasm
Smad3
Akt
P
Ser473
Canonical TGFβ signaling
Non-canonical TGFβ signaling
Transcription
Protein synthesis
Myog
Mymx/Mymk
Spsb1
Myh1/3/7
Nucleus
Myoblast fusion and myogenic differentiation
Myoblasts
Maintenance of muscle homeostasis
Myotubes
TNF ↑
IL-1β ↑
IL-6↑
IL-1R
Gp130
Il6st siRNA
JAK2
STAT3
AG490
C188-9
NF-κB
BMS-345541
SPSB1
ITD-1
Disturbed muscle homeostasis

Figure S19
